# *Blumea balsamifera BbHDA6* regulates abiotic stress responses and flavonoid biosynthesis in transgenic plants

**DOI:** 10.3389/fpls.2026.1811058

**Published:** 2026-04-22

**Authors:** Qiumei Luo, Sihong Sang, Yuchen Liu, Kailang Mu, Shan Sha, Fei Ran, Changmao Guo, Shi Yao, Tianjian Wang, Zhengwei Zhang, Yuan Yuan, Yuxin Pang

**Affiliations:** 1 School of Pharmacy, Guizhou University of Traditional Chinese Medicine, Guiyang, China; 2 School of Biosciences and Biopharmaceutics, Guangdong Pharmaceutical University, Guangzhou, China

**Keywords:** abiotic stress response, flavonoid biosynthesis, functional validation, heterologous expression, histone deacetylase, transcriptome analysis

## Abstract

**Introduction:**

Flavonoids in the leaves of *Blumea balsamifera* (L.) DC. have prominent anti-inflammatory and antioxidant activities, conferring high medicinal value to the plant. However, the key genes governing flavonoid biosynthesis and environmental adaptability in this species remain uncharacterized. This study aimed to clarify the functions of *BbHDA6* (a histone deacetylase gene) in *B. balsamifera* flavonoid biosynthesis and abiotic stress responses.

**Methods:**

We cloned the full-length *BbHDA6* gene (1350 bp) and systematically analyzed its molecular characteristics and expression patterns. Heterologous functional validation was subsequently conducted in *Arabidopsis thaliana* and *Nicotiana benthamiana*. Transgenic *Arabidopsis* plants overexpressing *BbHDA6* were subjected to ABA, osmotic, and salt stress treatments, and the gene’s role in abiotic stress responses was characterized by combining phenotypic analysis with transcriptome sequencing. Meanwhile, the function of *BbHDA6* in flavonoid biosynthesis was investigated in transgenic plants.

**Results:**

Results showed that *BbHDA6* localizes to the nucleus, is predominantly expressed in the upper leaves of *B. balsamifera*, and responds to abiotic stress. Overexpression of *BbHDA6* in transgenic *Arabidopsis* enhanced plant tolerance to low ABA concentrations but increased sensitivity to high ABA concentrations. In addition, *BbHDA6* regulated salt stress responses in a developmental stage-dependent manner and positively improved osmotic stress tolerance. Transcriptome analysis of *BbHDA6*-transgenic *Arabidopsis* revealed that *BbHDA6* modulates key abiotic stress-responsive genes and acts as a negative regulator of flavonoid biosynthetic genes. Furthermore, overexpression of *BbHDA6* in *N. benthamiana* reduced the total leaf flavonoid content by 29.72%–37.18%.

**Discussion:**

Collectively, this study is the first to identify *BbHDA6* as an epigenetic factor regulating abiotic stress resistance and flavonoid biosynthesis in *B. balsamifera*, providing a key molecular target for the genetic improvement of plant flavonoid content and stress-resistant traits.

## 1 Introduction

In natural habitats, plants constantly face challenges from various abiotic stresses such as drought, salinity, and extreme temperatures, which severely restrict their geographical distribution, growth and development, as well as agricultural productivity (Zhu, 2016). To cope with these environmental pressures, plants have evolved a complex and sophisticated regulatory network involving multi−level responses ranging from stress perception and signal transduction to reprogramming of physiological metabolism and developmental processes (Gechev and Hille, 2012; Haak et al., 2017; Kim et al., 2024; Kumar et al., 2019; Tuteja and Sopory, 2008). In recent years, epigenetic regulation, especially histone modification, has been demonstrated to play a crucial role in this process. By dynamically altering chromatin status, it flexibly and reversibly regulates gene expression without changing the DNA sequence, thereby conferring plants with plasticity to adapt to environmental changes (Chang et al., 2020; Shi et al., 2024). Among these regulators, histone deacetylases (HDACs) act as important epigenetic “erasers”. They catalyze deacetylation of lysine residues at the N−terminus of histones, induce chromatin condensation, and consequently repress gene transcription, making them key epigenetic regulators of plant development, metabolism, and stress adaptation (Vincent et al., 2022).

In model plants such as *Arabidopsis thaliana*, the function of histone deacetylase 6 (HDA6) has been extensively studied. It has been confirmed to participate in the silencing of ribosomal RNA genes, leaf senescence, flowering time regulation, and responses to abscisic acid (ABA), salt, and drought stresses (Luo et al., 2012; Saradadevi et al., 2023; Wu et al., 2008; Xu et al., 2024). Recent studies have also shown that HDACs play important roles in the biosynthesis of secondary metabolites, including flavonoids (Huang et al., 2025; Su et al., 2021; Zeng et al., 2025). However, most current studies focus on model plants. The specific functions of HDA6 homologs in medicinal plants with important economic value remain largely unknown, especially regarding their roles in regulating the biosynthesis of pharmacologically active secondary metabolites (e.g., flavonoids) and their crosstalk with abiotic stress responses. This knowledge gap limits our potential to improve stress resistance and active ingredient content in medicinal plants through epigenetic engineering.

*Blumea balsamifera* (L.) DC. is a traditional and economically important medicinal plant in China. Its leaves are rich in bioactive secondary metabolites including flavonoids and volatile oils. Among them, flavonoids such as quercetin, luteolin, and blumeatin have been verified to exhibit significant superoxide anion scavenging activity, DPPH antioxidant activity, and xanthine oxidase (XO) inhibitory activity (Fazilatun et al., 2005; Pang et al., 2014). These activities endow *B. balsamifera* with diverse pharmacological effects including anti−inflammatory, antioxidant, and antimicrobial properties, supporting its wide application and high market demand in the pharmaceutical industry. Flavonoids not only serve as the material basis for the core medicinal effects of *B. balsamifera*, but their biosynthetic pathway (the phenylpropanoid pathway) is also recognized as one of the central defensive metabolic networks for plants to integrate biotic and abiotic stress responses (Aluko et al., 2025; Dong and Lin, 2021; La Camera et al., 2004). This suggests that regulatory genes related to flavonoid biosynthesis may participate in the coordinated regulation of both medicinal metabolite accumulation and environmental adaptation. Meanwhile, multiple recent studies have confirmed that the RPD3/HDA1 subfamily, as a core group in plant epigenetic regulation, includes several members (e.g., AhHDA1 in peanut, GiHDA2b in licorice, and AtHDA19 in *Arabidopsis*) that regulate flavonoid biosynthesis through conserved histone deacetylation mechanisms (Huang et al., 2025; Su et al., 2021; Zeng et al., 2025). As a key member of this subfamily, HDA6 exhibits structural conservation and functional relevance, providing important theoretical support for predicting its potential involvement in flavonoid biosynthesis. However, it remains unclear whether the HDA6 homolog in *B. balsamifera* retains this conserved subfamily function, what its specific regulatory pattern and molecular mechanism may be, and whether it coordinately regulates flavonoid biosynthesis and abiotic stress responses.

To address the aforementioned research gaps, the present study was conducted using *B. balsamifera* as the experimental material to carry out a systematic investigation as follows: first, the *BbHDA6* gene was cloned, and its molecular characteristics and evolutionary conservation were elucidated through bioinformatic analyses; second, stable genetic transformation in *Nicotiana benthamiana* and *Arabidopsis* combined with subcellular localization assays was performed to uncover its regulatory function in flavonoid biosynthesis; third, its physiological functions in abiotic stress responses were explored under ABA, salt and mannitol stress treatments; finally, transcriptome sequencing was integrated to reveal the *BbHDA6*-mediated regulatory network underlying flavonoid biosynthesis and stress responses. This study aimed to characterize the basic molecular properties of *BbHDA6* and its regulatory role in flavonoid biosynthesis, as well as to clarify the regulatory patterns of *BbHDA6* in response to different abiotic stresses. This work is the first to uncover the function of an *HDA6* homolog in *B. balsamifera*, thereby providing novel candidate genes and a theoretical basis for the coordinated improvement of crop stress resistance and quality traits via epigenetic editing technologies.

## 2 Materials and methods

### 2.1 Extraction of total RNA from *Blumea balsamifera* and cDNA synthesis

Young leaves from tissue-cultured seedlings of *B. balsamifera* were harvested and ground in liquid nitrogen. Total RNA was extracted using a commercial RNA extraction kit. RNA integrity was verified by 1% agarose gel electrophoresis, and RNA concentration and purity were determined using a Nano-Drop^®^ ND-2000 UV-Vis spectrophotometer. First-strand cDNA was synthesized from total RNA using a reverse transcription kit and stored at -20 °C until use.

### 2.2 Cloning of the *BbHDA6* gene from *B. balsamifera* and construction of expression vector

Sequence-specific primers were designed based on the HDA6 homologous cDNA sequence identified from the *B. balsamifera* transcriptome database (PRJNA950886) (Zhan et al., 2023). Using cDNA from young *B. balsamifera* leaves as the template, PCR amplification was performed with 2×SuperNova PCR Mix (Dye) high-fidelity DNA polymerase (Genstar, Beijing Kangrunye Biotechnology Co., Ltd.). The amplified products were detected by 1% agarose gel electrophoresis, and the target fragment was recovered by gel extraction. After PCR amplification, ligation, transformation, and sequencing, the coding region of *BbHDA6* was verified using the NCBI ORF Finder online tool (https://www.ncbi.nlm.nih.gov/orffinder/). Finally, *BbHDA6* was cloned into the pBGOL-0017 vector, followed by transformation and further sequencing confirmation. The primers used for gene cloning and vector construction are listed in **Supplementary Table S1**.

### 2.3 Sequence analysis of *BbHDA6* from *B. balsamifera*

Bioinformatics analysis of the amino acid sequence and protein encoded by the *HDA6* gene was performed using online tools and software: the basic physicochemical properties were analyzed via ExPASy-ProtParam (https://web.expasy.org/protparam/); ProtScale (https://web.expasy.org/protscale/) was used for hydrophilicity/hydrophobicity analysis; subcellular localization was predicted with CELLO v.2.5 (https://cello.life.nctu.edu.tw/); SignalIP-4.1 (https://services.healthtech.dtu.dk/services/SignalP-4.1/) and TMHMM-2.0 (https://services.healthtech.dtu.dk/services/TMHMM-2.0/) were employed to predict signal peptides and transmembrane domains, respectively; the conserved functional domains were analyzed using the CDD tool in the NCBI database (https://www.ncbi.nlm.nih.gov/Structure/cdd/wrpsb.cgi); conserved motifs were analyzed through the MEME website (https://meme-suite.org/meme/tools/meme); homologous sequences of HDA6 proteins from different species were downloaded from NCBI (https://www.ncbi.nlm.nih.gov/orffinder/), a phylogenetic tree was constructed using MEGA-12 software, and homology alignment was performed with DNAMAN 7.0 software.

### 2.4 Subcellular localization of *BbHDA6* in *Arabidopsis* protoplasts

Using the recombinant plasmid pBG0017-*BbHDA6* as template, primers were designed based on restriction sites within the coding region for subcloning. After purification, the target fragment was inserted into the pUC19-GFP vector downstream of the 35S promoter using seamless cloning. The construct was transformed into Escherichia coli DH5α, verified by colony PCR and sequencing, and then used for midiprep. The pUC19-*BbHDA6*-GFP plasmid was co-transformed into *Arabidopsis* protoplasts together with the NLS-RFP plasmid (expressing a nuclear localization signal-tagged red fluorescent protein). After incubation in darkness at 25 °C, GFP fluorescence was observed under a laser scanning confocal microscope (TCS SP8, Leica, Germany) at 488 nm excitation and 546 nm emission wavelengths.

### 2.5 Tissue expression pattern analysis of *BbHDA6* in *B. balsamifera*

Primers were designed based on the cDNA sequence of *BbHDA6*, with EF1α-F/EF1α-R serving as the reference gene (Huang et al., 2024). Reverse transcription quantitative real-time PCR (RT-qPCR) was performed to analyze the tissue-specific expression pattern of *BbHDA6* in roots, stems, upper leaves, middle leaves, lower leaves and flowers of *B. balsamifera* plants at the flowering stage (upper leaves refer to the top 1~2 fully expanded young leaves, middle leaves are the 3rd to 5th fully expanded leaves from the top, and lower leaves are the leaves below the 5th node). The RT-qPCR assays were conducted using the 2×RealStar Fast SYBR qPCR Mix (Genstar, Beijing, China) on a Bio-Rad CFX96 Touch Real-Time PCR Detection System (Bio-Rad, California, USA). The 10 μL reaction system consisted of 3 μL of 10-fold diluted cDNA template, 5 μL of 2×RealStar Green Fast Mixture, 0.5 μL each of the forward and reverse primers, and 1 μL of RNase-free H_2_O. The amplification program was set as follows: pre-denaturation at 95 °C for 2 min; followed by 40 cycles of denaturation at 95 °C for 15 s, annealing at 57 °C for 30 s and extension at 72 °C for 30 s; with a final extension step at 72 °C for 5 min (fluorescence signals were collected throughout the process). Three biological replicates were set for each tissue sample. The 2^^-^ΔΔCt^ method was used to calculate the relative expression levels of *BbHDA6* in different tissues, and the expression histograms were generated using GraphPad Prism software.

### 2.6 Expression analysis of *BbHDA6* under abiotic stress treatments

Leaves of four-month-old tissue-cultured *B. balsamifera* seedlings grown in a greenhouse were subjected to four abiotic stress treatments with specific conditions as follows: 30% PEG (simulated drought stress, continuous treatment), 4°C low temperature (continuous low-temperature stress), 200 mM NaCl (salt stress, continuous treatment), and 200 μM ABA (hormone stress, continuous treatment). All stress treatments were conducted continuously until the end of sampling. Leaf samples were collected at 0 h (control, before stress treatment), 6 h, 12 h, 24 h and 48 h post the initiation of stress treatment, immediately snap-frozen in liquid nitrogen, and stored at -80 °C for subsequent analysis. Total RNA was extracted from the treated leaf samples, and the synthesized cDNA via reverse transcription was used as the template to determine the expression level of BbHDA6 by RT-qPCR. The reference gene, PCR reaction system and amplification program used in this assay were identical to those described in Section 2.5 (**Supplementary Table S1**). Three biological replicates were set for each stress treatment and each sampling time point.

### 2.7 Transformation and functional validation in *Arabidopsis*

#### 2.7.1 Plant material preparation

Wild-type *Arabidopsis* seeds were vernalized at 4°C for 4 days, then sown on vermiculite and incubated under moist conditions with a cover for 5 days. The cover was subsequently removed, and the seedlings were cultured at 25 °C under a 16 h light/8 h dark photoperiod with regular watering and nutrient supplementation for subsequent experiments.

#### 2.7.2 Generation of transgenic plants and RT-qPCR analysis

*Arabidopsis* was genetically transformed via the floral dip method, with three groups set up: the experimental group (*BbHDA6*-transformed), and the control groups including the wild-type (WT) and empty vector (EV)-transformed lines. T1 transgenic plants were cultured in soil for screening; after two cotyledons emerged, the leaves were sprayed with a 600 mg/L kanamycin (Kana) solution to select positive plants, with the spraying conducted once every 2 days for a total of six times. Surviving plants were verified for successful transformation by PCR. Seeds of the T2 and T3 generations were screened on antibiotic-containing plates with 30 mg/L Kana. The expression levels of *BbHDA6* in five transgenic *Arabidopsis* lines (OE-3, OE-4, OE-7, OE-9, OE-11) were determined by RT-qPCR. One high-expression line and one low-expression line were selected for subsequent functional validation assays.

#### 2.7.3 Abiotic stress responses of *BbHDA6*-overexpressing *Arabidopsis*

##### 2.7.3.1 Seed germination assay under abiotic stresses

Seeds of wild-type *Arabidopsis*, empty vector pBGOL-0017-transformed (EV) line and *BbHDA6*-overexpressing transgenic lines were incubated at 4°C for 4 days and subjected to surface sterilization, then sown separately on media supplemented with 0, 0.5, 1, 5 μM ABA, 100, 200, 300 mM mannitol, or 100, 150, 200 mM NaCl, respectively. All seeds were cultured at 25 °C under a 16 h light/8 h dark photoperiod, and the seed germination rate was recorded daily for 7 consecutive days, with germination defined as radicle length exceeding half the seed length. Photographs of the seed germination phenotype were taken at 7 days growth on media (i.e., at the end of the 7-day germination recording period). Three biological replicates were performed for the assay, with approximately 50 seeds sown per replicate.

##### 2.7.3.2 Root elongation assay under abiotic stresses

Seeds of wild-type (WT) *Arabidopsis*, pBGOL-0017 empty vector (EV)-transformed line and *BbHDA6*-overexpressing transgenic lines were surface-sterilized and then sown on 1/2 MS medium, followed by vertical culture at 25 °C under a 16 h light/8 h dark photoperiod for 5 days. Seedlings of different genotypes with uniform root growth were selected and transferred to fresh media supplemented with 0, 0.5, 1, 5 μM ABA, 100, 200, 300 mM mannitol, or 100, 150, 200 mM NaCl. After another 10 days of vertical culture, the root lengths of the seedlings were measured.

##### 2.7.3.3 Stress sensitivity assay

Five-day-old seedlings of wild-type (WT) *Arabidopsis*, pBGOL-0017 empty vector (EV)-transformed line, and *BbHDA6*-transformed transgenic line were transplanted separately onto media supplemented with 0, 0.5, 1, 5 μM ABA, 100, 200, 300 mM mannitol, or 100, 150, 200 mM NaCl, and cultured for 7 days. The growth status of the seedlings was observed and recorded on the 7th day of culture. Stress survival analysis was carried out as described by Chen et al (Chen et al., 2010).

#### 2.7.4 RNA−seq analysis of *BbHDA6*-overexpressing *Arabidopsis* under abiotic stress

##### 2.7.4.1 Plant material and treatment

Four-week-old wild-type (WT) *Arabidopsis* plants and two transgenic lines (high-expression line OE-3 and low-expression line OE-9) with uniform growth status were selected. The selected plants were immersed in 200 μM ABA or 200 mM NaCl solution for 6 h, while the control group was cultured under normal conditions without any treatment; all samples were used for transcriptome sequencing. Total RNA of each sample was extracted using the MJZol Total RNA Extraction Kit (Meiji Biomedical Technology Co., Ltd., Shanghai, China). RNA quality and concentration were determined with an Agilent 2100 Bioanalyzer (Agilent Technologies, Waldbronn, Germany). RNA-seq libraries were constructed following the protocol of the Illumina^®^ Stranded mRNA Prep, Ligation Kit (San Diego, California, USA). Three biological replicates were set for each group.

##### 2.7.4.2 Transcriptome data analysis

cDNA synthesis, library construction, sequencing, and raw read analysis were performed as previously described (Yang et al., 2017). Clean reads were aligned to the reference *Arabidopsis* genome using TopHat2 (Kim et al., 2013). Gene expression was quantified via the RPKM method (Mortazavi et al., 2008). Differentially expressed genes (DEGs) between genotypes and treatments were identified using DESeq2 (Pang et al., 2024); genes with FDR < 0.05 and |log2(fold change)| ≥ 1 were designated as DEGs. GO enrichment analysis was conducted using the agriGO tool (Tian et al., 2017), and functional annotation of DEGs was performed using the GO (Gene Ontology, http://www.geneontology.org/) and KEGG (Kyoto Encyclopedia of Genes and Genomes, http://www.genome.jp/kegg/) databases. RNA-seq data have been submitted to NCBI (SRA accession number: PRJNA1421045).

### 2.8 Determination of total flavonoid content and key gene expression in *BbHDA6*-transgenic *N. benthamiana*

In this study, *Agrobacterium*-mediated leaf disc transformation was used to achieve stable transformation of the *BbHDA6* gene into *Nicotiana benthamiana*. Two homozygous T3-generation *BbHDA6*-transgenic *N. benthamiana* lines (*BbHDA6*-OE-2 and *BbHDA6*-OE-5) were screened and cultured for functional validation assays. Wild-type (WT) *N. benthamiana* and the two transgenic lines (*BbHDA6*-OE-2 and *BbHDA6*-OE-5) grown for 4 weeks under a 16 h light/8 h dark photoperiod were selected to detect the expression levels of *BbHDA6* and seven key genes involved in the flavonoid biosynthetic pathway. The PCR system and amplification program were identical to those described in Section 2.5. The sodium nitrite-aluminum nitrate spectrophotometric method was employed to determine the total flavonoid content in the leaves of WT and the two transgenic *N. benthamiana* lines. The primers used for the reference genes and key flavonoid biosynthetic genes in *N. benthamiana* are listed in **Supplementary Table S2**. Three biological replicates were set for all experiments.

### 2.9 Statistical analysis

Statistical analysis of experimental data was performed using SPSS 21.0 and Excel 2021 software, and graphs were generated using GraphPad Prism 9.5 and OriginPro 2024 software. All data were presented as the mean ± standard deviation (mean ± SD) of three biological replicates. Analysis of variance (ANOVA) and non-parametric tests were employed to assess the significance of differences between datasets.

## 3 Results

### 3.1 Identification and bioinformatics analysis of *BbHDA6*

**Figure 1:**
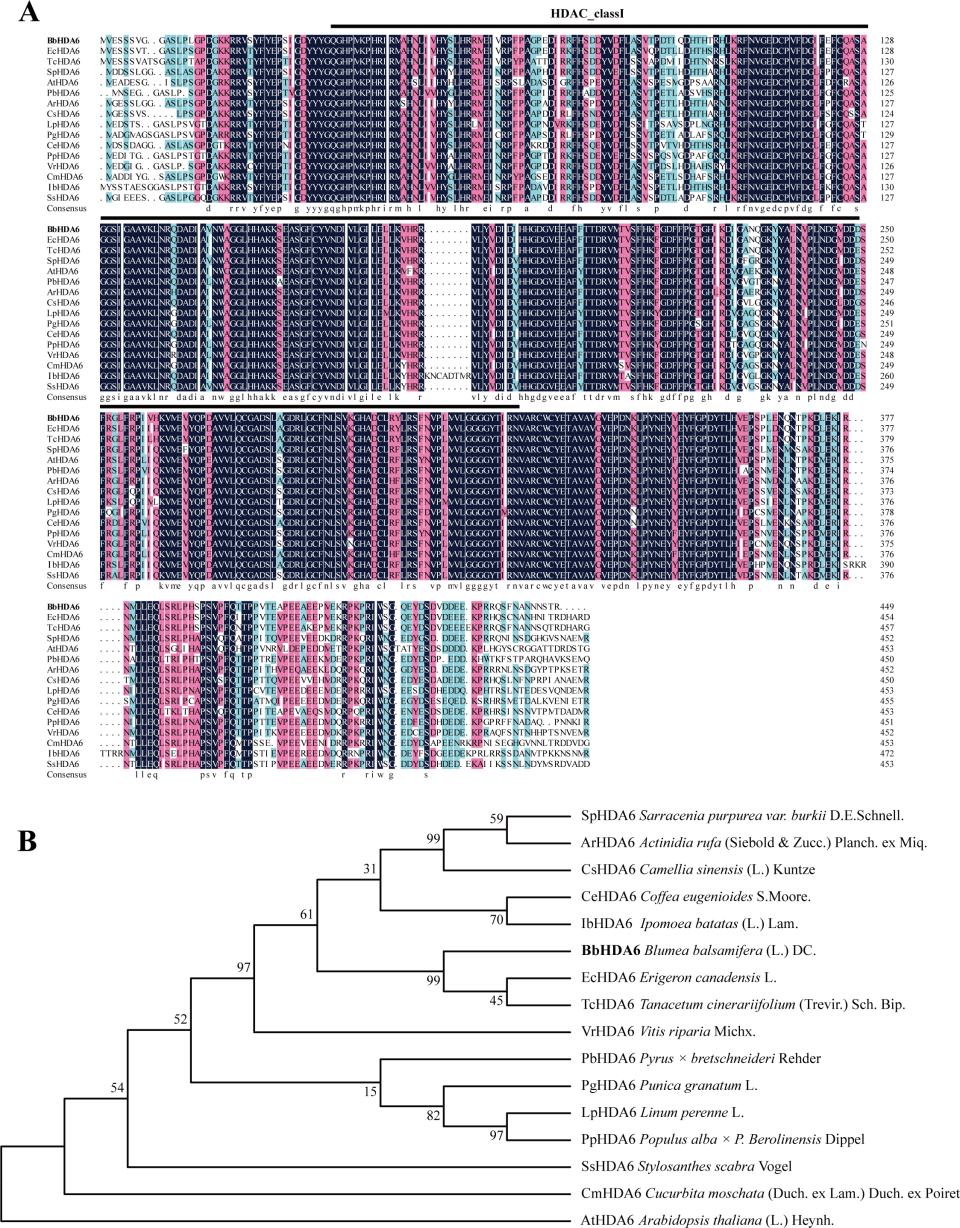
Alignment of homologous sequences and phylogenetic tree analysis of BbHDA6. **(A)** Alignment of homologous sequences; **(B)** Phylogenetic tree analysis. The HDA6 homologous proteins employed in the sequence alignment and phylogenetic analysis are listed below. *Sarracenia purpurea* var. *burkii* D.E.Schnell: SpHDA6 (KAL6973176.1); *Actinidia rufa* (Siebold & Zucc.) Planch. ex Miq: ArHDA6 (GFZ12010.1); *Camellia sinensis* (L.) Kuntze: CsHDA6 (XP_028054450.1); *Coffea eugenioides* S.Moore: CeHDA6 (XP_027169164.1); *Ipomoea batatas* (L.) Lam: IbHDA6 (GMD36299.1); *Erigeron canadensis* L: EcHDA6 (XP_043607769.1); *Tanacetum cinerariifolium* (Trevir.) Sch. Bip: TcHDA6 (GEV93100.1); *Vitis riparia* Michx: VrHDA6 (XP_034673356); *Pyrus × bretschneideri* Rehder: PbHDA6 (XP_048445786.1); *Punica granatum* L: PgHDA6 (XP_031371384.1); *Linum perenne* L: LpHDA6 (CAN1294289.1); *Populus alba × P. Berolinensis* Dippel: PpHDA6 (KAJ6970579.1); *Stylosanthes scabra* Vogel: SsHDA6 (MED6126235.1); *Cucurbita moschata* (Duch. ex Lam.) Duch. ex Poiret: CmHDA6 (XP_022922101.1); *Arabidopsis thaliana* (L.) Heynh: AtHDA6 (AT5G63110).

The histone deacetylase gene homologous to *HDA6* was isolated from the *B. balsamifera* transcriptome database (PRJNA950886) via sequence alignment and named *BbHDA6*. The CDS of *BbHDA6* is 1350 bp in length (**Supplementary Figure 1**), encoding 449 amino acids (GenBank: PX898059). According to bioinformatic prediction, *BbHDA6* is an acidic, unstable, hydrophobic protein without signal peptides or transmembrane domains (**Supplementary Figure 2**), and is localized to the nucleus. Conserved domain and BLAST analysis showed it contains a typical Class I HDAC domain (RPD3/HDA1 subfamily histone deacetylase) and exhibits high sequence similarity to HDA6 homologs from *Arabidopsis thaliana* (74.42% similarity to AtHDA6) and other plant species (**Figure 1A**). Phylogenetic analysis revealed BbHDA6 is most closely related to EcHDA6 (*Erigeron canadensis*) and TcHDA6 (*Tanacetum cinerariifolium*), both of which belong to the Asteraceae family (**Figure 1B**). And conserved motifs analysis showed highly similar distributions of the ten motifs among BbHDA6, AtHDA6, EcHDA6 and TcHDA6 (**Supplementary Figure 3**). These results suggest the functional conservation of *BbHDA6* in *B. balsamifera*.

### 3.2 Subcellular localization and expression pattern of *BbHDA6*

**Figure 2:**
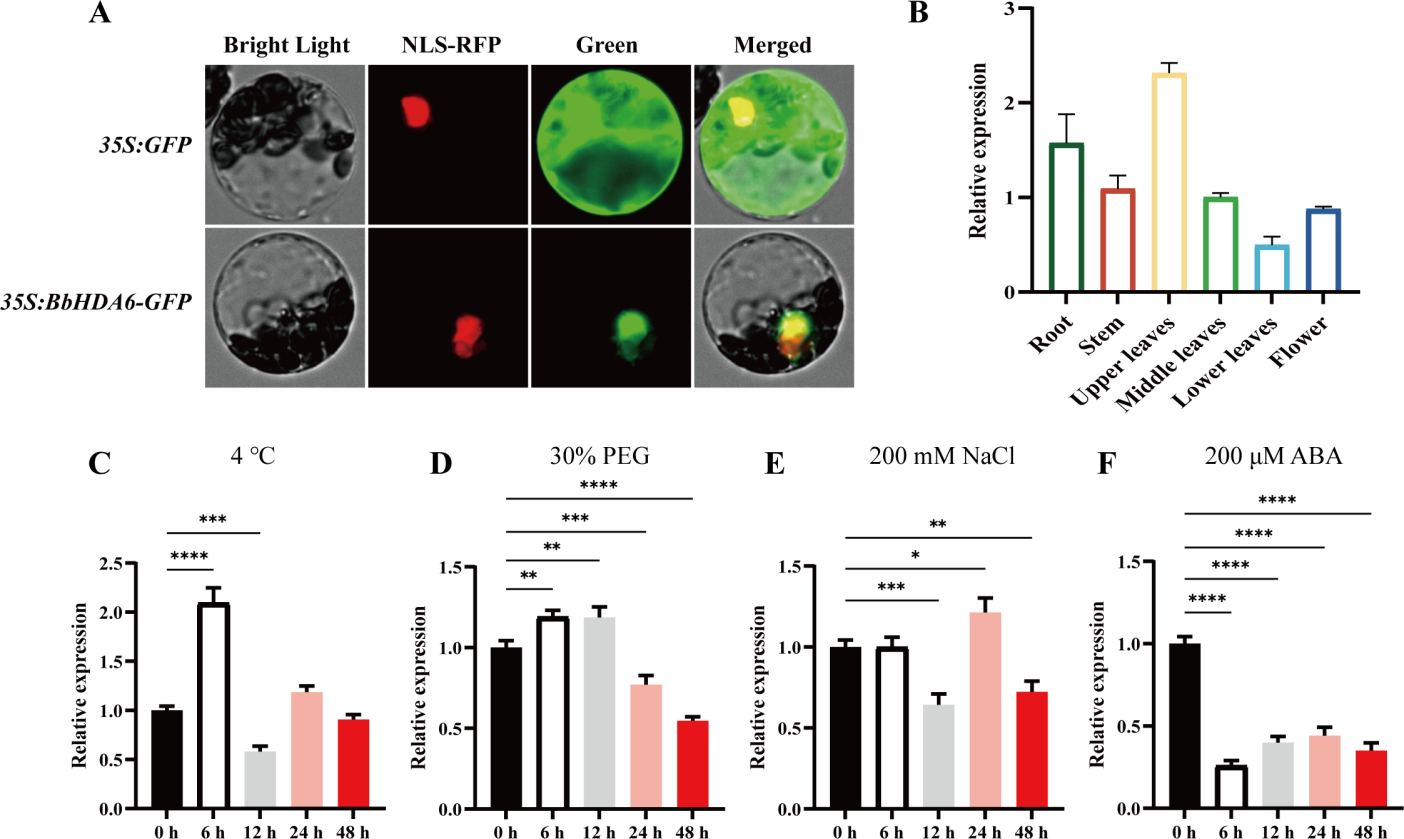
Subcellular Localization of *BbHDA6* in *Arabidopsis thaliana* Protoplasts and Analysis of Its Expression Pattern in *Blumea balsamifera* Leaves. **(A)** Subcellular localization results. NLS-RFP: Nuclear Localization Signal – Red Fluorescent Protein, a nuclear localization signal-tagged red fluorescent protein used as the nuclear marker; **(B)** Expression levels of *BbHDA6* in different tissues; **(C–F)** Expression levels of *BbHDA6* in *B. balsamifera* leaves under different abiotic stresses and ABA treatment. Specific treatment conditions were as follows: **(C)** 4 °C low temperature stress (continuous treatment), **(D)** 30% PEG (simulated drought stress, continuous treatment), **(E)** 200 mM NaCl (salt stress, continuous treatment), **(F)** 200 μM ABA (hormone stress, continuous treatment). All treatments were conducted continuously, and leaf samples were collected at 0 h (control, before treatment initiation), 6 h, 12 h, 24 h, and 48 h post the start of treatment to detect the *BbHDA6* expression levels. Statistical analysis was performed by One-way ANOVA followed by Dunnett’s multiple comparisons test. *P < 0.05, **P < 0.01, ***P < 0.001, ****P < 0.0001.

Subcellular localization analysis confirmed the nuclear localization of *BbHDA6*, consistent with prior bioinformatics prediction (**Figure 2A**). qRT−PCR analysis revealed that *BbHDA6* exhibited tissue−specific expression in *B. balsamifera*, with the highest expression in upper leaves, followed by roots, stems, middle leaves, and flowers, and the lowest in lower leaves (**Figure 2B**). To investigate the role of *BbHDA6* in abiotic stress responses, its expression level was examined under various abiotic stress treatments. Low temperature (4°C) and PEG-simulated drought (30% PEG) significantly upregulated *BbHDA6* expression within 6 hours (**Figures 2C, D**). Under salt stress, its transcription was first downregulated and then peaked at 24 h (**Figure 2E**). Under abscisic acid (ABA) treatment, *BbHDA6* expression was significantly repressed compared with that at 0 h (**Figure 2F**). These results demonstrate that BbHDA6 localizes and functions in the nucleus and responds to 4°C low temperature, drought, salt, and ABA stresses, laying a foundation for further elucidating its regulatory mechanism in plant stress adaptation.

### 3.3 *BbHDA6 affects* ABA responses in transgenic *Arabidopsis*

**Figure 3:**
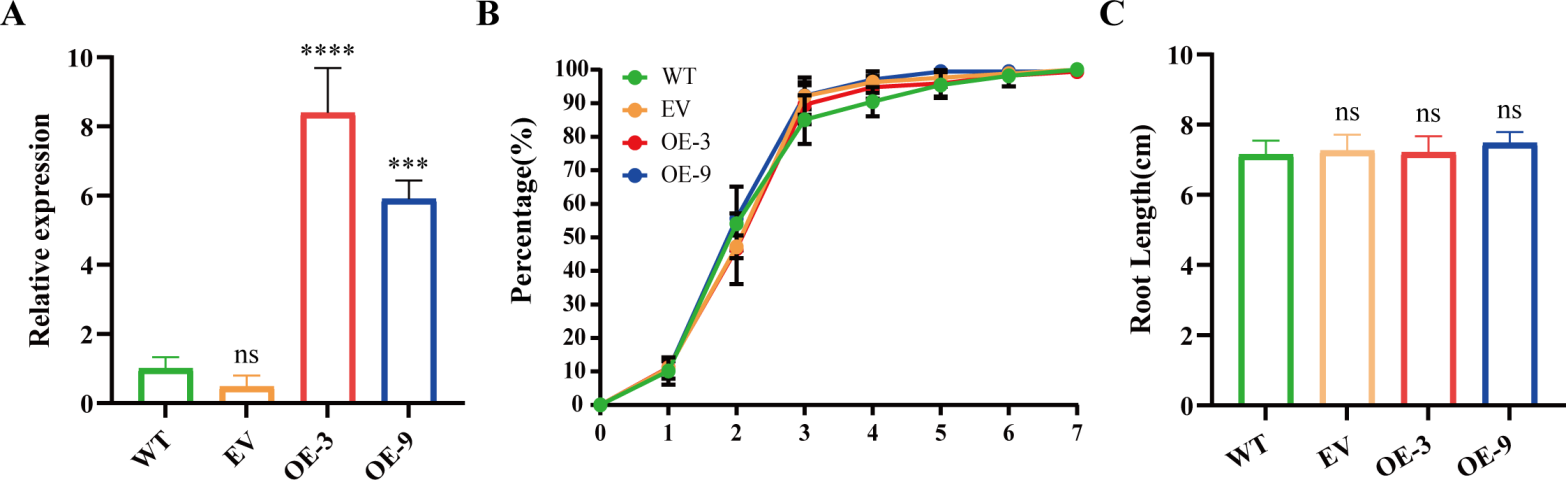
Phenotypic analysis of *BbHDA6* transgenic Arabidopsis thaliana on 1/2 MS medium. (A) Expression levels of *BbHDA6* in transgenic lines OE-3 and OE-9. One-way ANOVA followed by Dunnett’s test was used to compare *BbHDA6* expression levels between *BbHDA6*-transgenic, empty vector (pBGOL-0017) transgenic, and wild–type (WT) *Arabidopsis*. ns: Not statistically significant (*P* > 0.05), ***P < 0.001, ****P < 0.0001; (B) Seed germination rates of WT, EV, OE-3, and OE-9 on 1/2 MS medium for 7 consecutive days. The color coding in these figures represents different *Arabidopsis* genotypes. Statistical analysis was performed by two–way ANOVA followed by Dunnett’s multiple comparisons test; (C) Root elongation phenotypes of WT, EV, OE-3, and OE-9 cultured on 1/2 MS medium for 10 days. One-way ANOVA followed by Dunnett’s test was used. *P* < 0.05, ns: Not statistically significant (*P* > 0.05).

**Figure 4:**
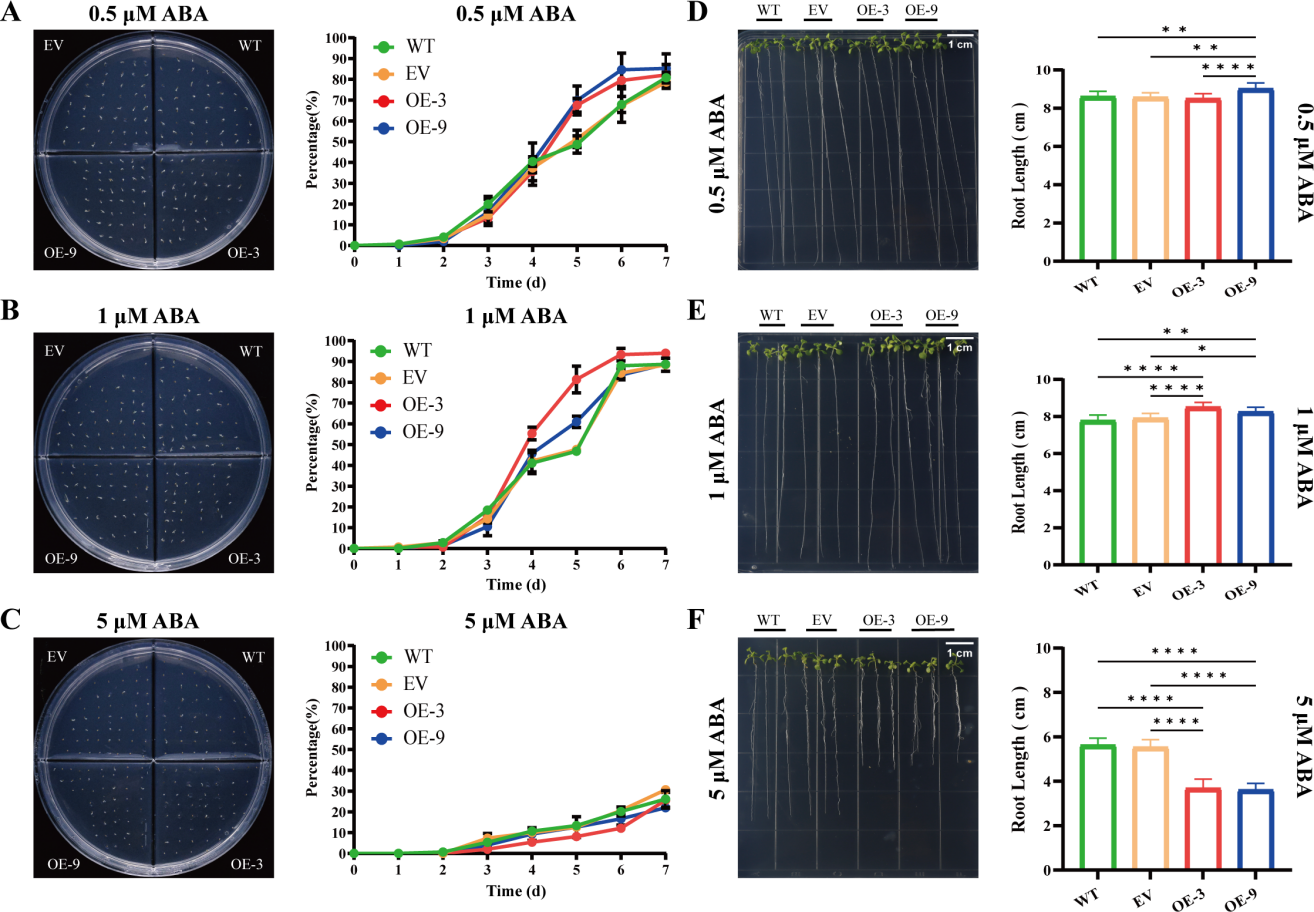
Phenotypic analysis of *BbHDA6*-transgenic *Arabidopsis thaliana* on ABA stress media. **(A–C)** Seed germination dynamics of *BbHDA6*-transgenic *Arabidopsis* for 7 consecutive days under 0.5, 1, and 5 μM ABA treatments, respectively. Photographs of germination phenotypes for WT, EV, OE-3, and OE-9 seeds were taken at 7 days after growth under ABA treatments. Statistical analysis was performed by two-way ANOVA followed by Dunnett’s multiple comparisons test. **(D–F)** Root growth phenotypes of *BbHDA6*-transgenic *Arabidopsis* after 10 days of culture under 0.5, 1, and 5 μM ABA treatments, respectively. Photographs of root growth phenotypes for WT, EV, OE-3, and OE-9 seedlings were taken at 10 days after culture under ABA treatments. One-way ANOVA followed by Tukey’s test was used to analyze the significance of differences in root length under 0.5 and 5 μM ABA treatments, while the Kruskal-Wallis test followed by Dunn’s multiple comparisons test was applied for root length under 1 μM ABA treatment. *P < 0.05, **P < 0.01, ****P < 0.0001.

The role of *BbHDA6* in ABA response was further investigated in transgenic *Arabidopsis*. Two transgenic lines (OE-3 and OE-9) with relatively high and low expression levels of *BbHDA6* were used for phenotypic analysis (**Figure 3A**; **Supplementary Figure 4**). Under normal conditions, there were no significant differences in seed germination or root elongation between transgenic plants and the wild−type (WT) (**Figures 3B, C**; **Supplementary Table S3**). When treated with 0.5 μM ABA, both transgenic lines showed higher germination rates than WT from days 5 to 7, and roots of OE-9 were significantly longer than those of WT (**Figures 4A, D**; **Supplementary Table S4**). Under 1 μM ABA treatment, the germination rate of OE-3 was higher than that of WT from day 4, while OE-9 was higher only on day 5; both transgenic lines exhibited significantly longer roots than WT (**Figures 4B, E**; **Supplementary Table S5**). Germination of transgenic lines and WT was significantly inhibited under treatment with a high ABA concentration (5 μM). Germination of OE−3 decreased markedly from days 4 to 6, and seedlings of both transgenic lines had shorter roots than WT (**Figures 4C, F**; **Supplementary Table S6**). In the survival rate assay, no plant death or abnormal growth was observed in either the 1/2 MS medium group or any ABA treatment group (**Supplementary Figure 5A**). These results suggested that *BbHDA6* enhances tolerance to low ABA concentrations (0.5 and 1 μM) and increases sensitivity to high ABA concentration (5 μM) in transgenic *Arabidopsis*. Thus, *BbHDA6* mediates ABA responses and participate in the ABA signaling pathway.

### 3.4 *BbHDA6* is involved in the osmotic stress response of transgenic *Arabidopsis*

**Figure 5:**
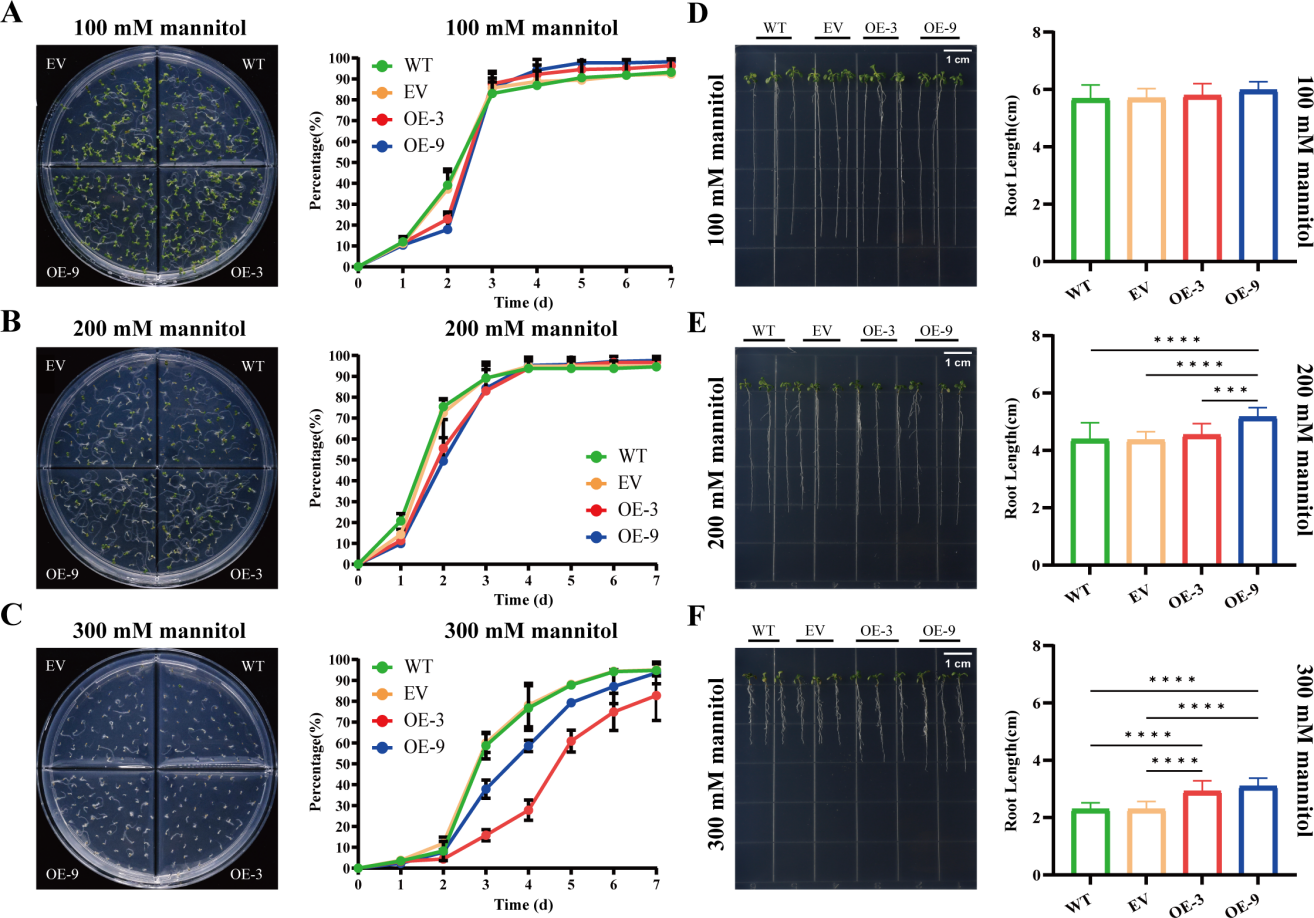
Phenotypic analysis of *BbHDA6*-transgenic *Arabidopsis thaliana* on mannitol stress media. **(A–C)** Seed germination dynamics of *BbHDA6*-transgenic *Arabidopsis* for 7 consecutive days under 100, 200, and 300 mM mannitol treatments, respectively. Photographs of germination phenotypes for WT, EV, OE-3, and OE-9 seeds were taken at 7 days after growth under mannitol treatments. Statistical analysis was performed by two-way ANOVA followed by Dunnett’s multiple comparisons test. **(D–F)** Root growth phenotypes of *BbHDA6*-transgenic *Arabidopsis* after 10 days of culture under 100, 200, and 300 mM mannitol treatments, respectively. Photographs of root growth phenotypes for WT, EV, OE-3, and OE-9 seedlings were taken at 10 days after culture under mannitol treatments. One-way ANOVA followed by Tukey’s test was used to analyze the significance of differences in root length under 200 and 300 mM mannitol treatments, while the Kruskal-Wallis test followed by Dunn’s multiple comparisons test was applied for root length under 100 mM mannitol treatment. ***P < 0.001, ****P < 0.0001.

To investigate the role of *BbHDA6* in osmotic stress responses, this study analyzed the seed germination rate, root length, and stress survival rate of *BbHDA6*-transgenic *Arabidopsis* under mannitol treatment. The results of seed germination statistics showed that under 100 mM mannitol treatment, the two transgenic lines (high-expression line OE-3 and low-expression line OE-9) had lower seed germination rates than the wild type (WT) on day 2, but higher germination rates than WT from days 4 to 7 (**Figure 5A**; **Supplementary Table S7**). Under 200 mM mannitol treatment, the two transgenic lines exhibited lower seed germination rates than WT on days 1~3, and higher germination rates than WT starting from day 5 (**Figure 5B**; **Supplementary Table S8**). Under high-concentration mannitol (300 mM) treatment, the germination rates of the two transgenic lines were lower than that of WT from day 2 onward; among them, the high-expression line OE-3 consistently had lower germination rates than the low-expression line OE-9 from day 2 (**Figure 5C**; **Supplementary Table S9**). Further measurement of the root length of seedlings grown on medium containing different concentrations of mannitol showed that overexpression of *BbHDA6* promoted root elongation in transgenic *Arabidopsis* under all tested mannitol concentrations (100, 200, and 300 mM) (**Figures 5D–F**). In the survival rate assay, there was no significant difference in the number of abnormally growing plants (such as wilting and leaf chlorosis) among all groups under 100 mM mannitol treatment. Under 200 mM mannitol treatment, the number of abnormally growing plants in the two transgenic lines was significantly lower than that in WT. Under 300 mM mannitol treatment, OE-3 had significantly fewer abnormally growing plants than WT, while OE-9 had more (**Supplementary Figure 5B**). In summary, overexpression of *BbHDA6* enhanced the osmotic stress tolerance of *Arabidopsis* under 200 mM mannitol treatment. Under high-concentration mannitol treatment, overexpression of *BbHDA6* inhibited seed germination but promoted seedling root elongation in transgenic *Arabidopsis*. The survival rate results indicated that the higher the expression level of *BbHDA6* in transgenic *Arabidopsis*, the more stable the plant growth. Collectively, these findings demonstrate that *BbHDA6* is involved in the response of *Arabidopsis* to osmotic stress.

### 3.5 *BbHDA6* affects salt stress responses in *Arabidopsis*

**Figure 6:**
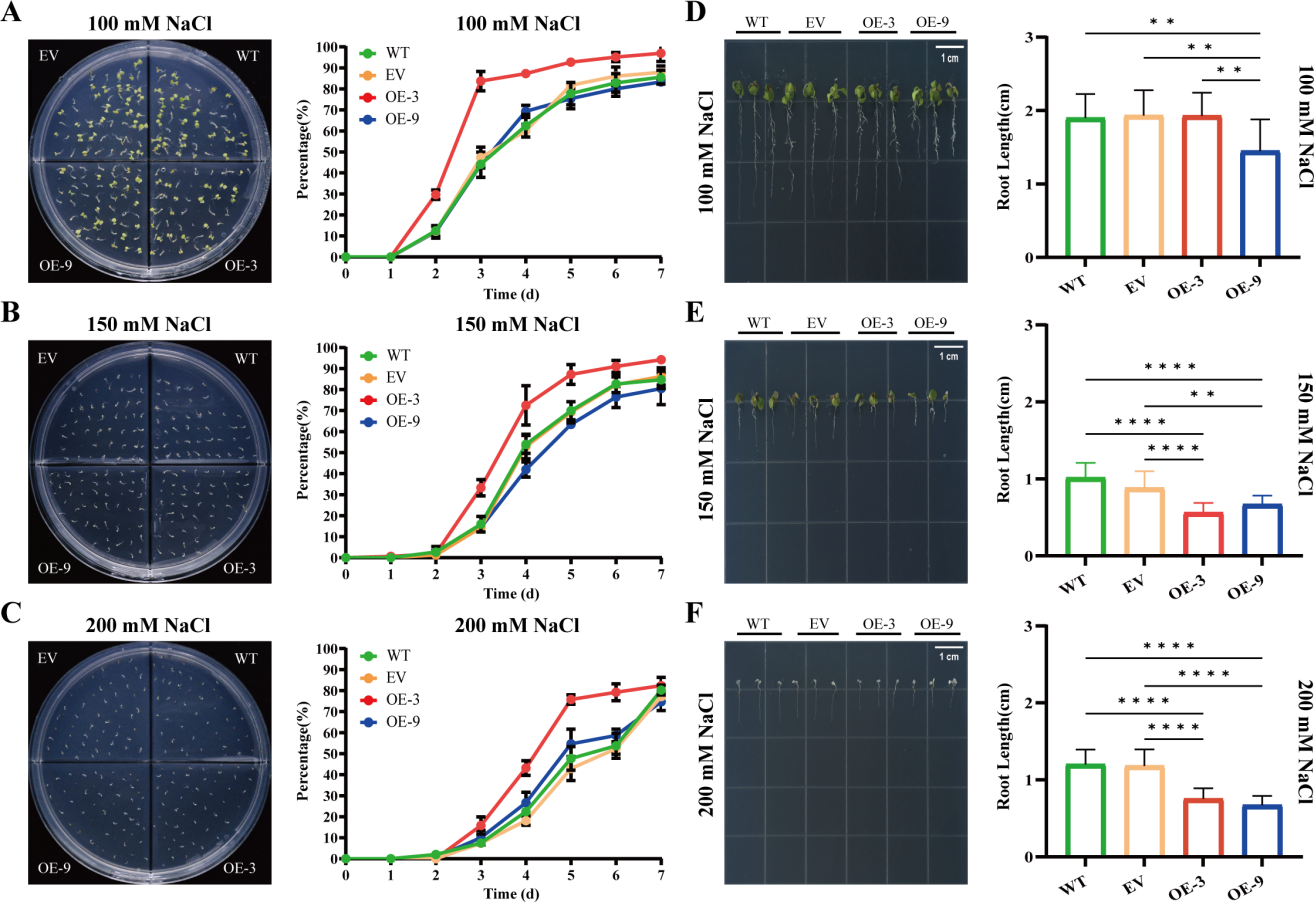
Phenotypic analysis of *BbHDA6*-transgenic *Arabidopsis thaliana* on NaCl stress media. **(A-C)** Seed germination dynamics of *BbHDA6*-transgenic *Arabidopsis* for 7 consecutive days under 100, 150, and 200 mM NaCl treatments, respectively. Photographs of germination phenotypes for WT, EV, OE-3, and OE-9 seeds were taken at 7 days after growth under salt treatments. Statistical analysis was performed by two-way ANOVA followed by Dunnett’s multiple comparisons test. **(D-F)** Root growth phenotypes of *BbHDA6*-transgenic *Arabidopsis* after 10 days of culture under 100, 150, and 200 mM NaCl treatments, respectively. Photographs of root growth phenotypes for WT, EV, OE-3, and OE-9 seedlings were taken at 10 days after culture under salt treatments. One-way ANOVA followed by Tukey’s test was used to analyze the significance of differences in root length under 150 mM NaCl treatment, while the Kruskal-Wallis test followed by Dunn’s multiple comparisons test was applied for root length under 100 and 200 mM NaCl treatments. **P < 0.01, ****P < 0.0001.

To evaluate whether *BbHDA6* mediates salt stress responses, we analyzed the seed germination rate and seedling root length of *BbHDA6*-transgenic *Arabidopsis* under salt treatment. When treated with 100 mM NaCl, OE-3 seed germination was consistently higher than WT and OE-9 from day 2, while OE-9 was only higher than WT on day 4; all genotypes had shorter roots compared with those under normal conditions, with OE-9 having the shortest roots; no plant death was observed, but all groups exhibited salt-sensitive phenotypes (leaf chlorosis, apical browning), and the proportion of plants with abnormal growth was higher in transgenic lines (57% for OE-3, 59% for OE-9) than the control group (44%) (**Figures 6A, D**; **Supplementary Figure 5C**; **Supplementary Table S10**). Under 150 mM NaCl treatment, the germination rate of OE-3 was higher than that of WT from day 3, while OE-9 was consistently lower from day 4; and both transgenic lines exhibited significantly shorter roots than WT (**Figures 6B, E**; **Supplementary Table S11**). Germination of transgenic lines and WT was significantly inhibited under treatment with a high salt concentration (200 mM NaCl), OE-3 showed a higher germination rate than WT from day 3, while OE-9 was higher from day 3 to 6 but lower on day 7; and both transgenic lines had significantly shorter roots than WT (**Figures 6C, F**; **Supplementary Table S12**). Collectively, overexpression of *BbHDA6* increased the seed germination rate of the high-expression line OE-3 under salt stress, while inhibiting root elongation of *BbHDA6*-transgenic *Arabidopsis* under 150~200 mM salt treatment, indicating that *BbHDA6* is involved in plant responses to salt stress.

### 3.6 *BbHDA6* regulates transcriptional reprogramming of genes in the ABA signaling pathway

**Figure 7:**
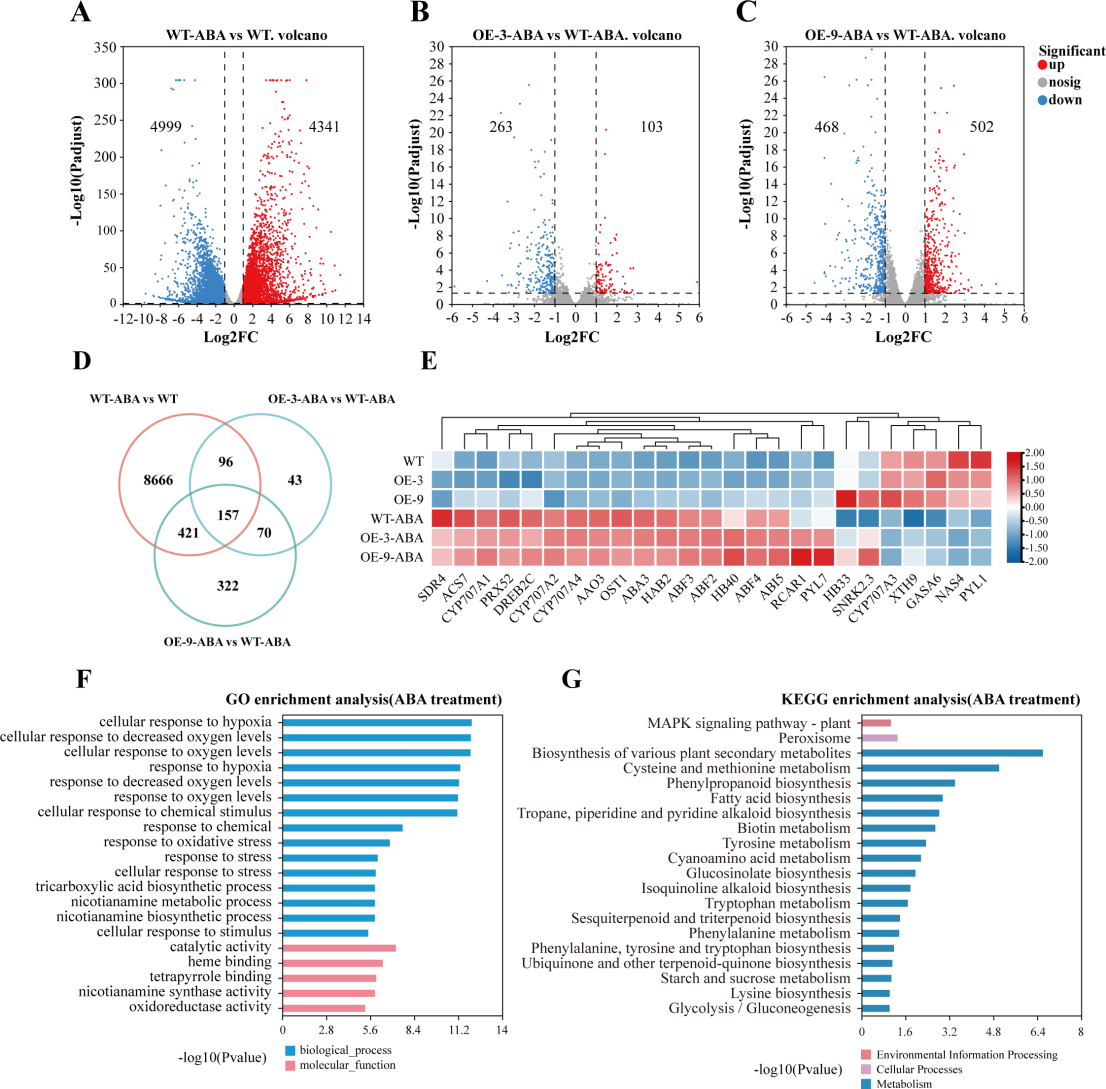
Transcriptome analysis of *BbHDA6* transgenic *Arabidopsis thaliana* under ABA stress. **(A–C)** Volcano plots of DEGs in transgenic *Arabidopsis* under ABA stress; **(D)** Venn analysis of *BbHDA6*-mediated DEGs in response to ABA stress; **(E)** Heatmap of *BbHDA6*-mediated ABA-responsive gene expression; **(F)** GO analysis of *BbHDA6*-mediated ABA-responsive genes; **(G)** KEGG analysis of *BbHDA6*-mediated ABA-responsive genes.

To investigate *BbHDA6*’s function in the ABA signaling pathway, we performed RNA-seq on transcripts from wild-type (WT) *Arabidopsis* and *BbHDA6*-transgenic lines (OE-3, OE-9) after ABA treatment. In WT, 9340 ABA stress-responsive genes were identified, with 1336 showing differential expression between WT and transgenic lines. Of these 1336 genes, 157 overlapped and were classified as *BbHDA6*-regulated ABA-responsive genes (**Figures 7A–D**). A heatmap of these genes showed 17.20% were positively regulated by *BbHDA6* and 82.80% negatively regulated. Gene Ontology (GO) enrichment analysis confirmed these genes were significantly enriched in biological processes such as cellular response to hypoxia and to chemical stimulus (**Figure 7F**). Kyoto Encyclopedia of Genes and Genomes (KEGG) enrichment analysis showed differentially expressed genes (DEGs) were enriched in pathways including plant secondary metabolite biosynthesis, cysteine and methionine metabolism, phenylpropanoid biosynthesis, fatty acid biosynthesis, tropane alkaloid biosynthesis, biotin metabolism, and tyrosine metabolism (**Figure 7G**). A heatmap of the 157 DEGs’ expression profiles is presented in **Supplementary Figure 6B**. Canonical ABA signaling pathway genes (*PYL1/7*, *RCAR1*, *HAB2*, *SnRK2.3*, *OST1*), core metabolic genes (*ABA3*, *AAO3*, *CYP707A1*, *CYP707A2*, *CYP707A3*, *CYP707A4*), downstream transcription factors (*ABI5*, *ABF2/3/4*, *DREB2C*), and stress-responsive genes (*HB33/40*, *GASA6*, *XTH9*, *NAS4*, *SDR4*, *ACS7*, *PRX52*) are well-known regulators of the ABA signaling response. These genes were differentially expressed between WT and the two transgenic lines after ABA treatment (**Figure 7E**), indicating *BbHDA6* participates in the ABA signaling response. Additionally, these genes regulate seed germination and root elongation, suggesting they are ABA signaling genes through which *BbHDA6* modulates seed germination and root growth.

### 3.7 *BbHDA6* regulates the expression of salt stress-responsive genes in *Arabidopsis*

**Figure 8:**
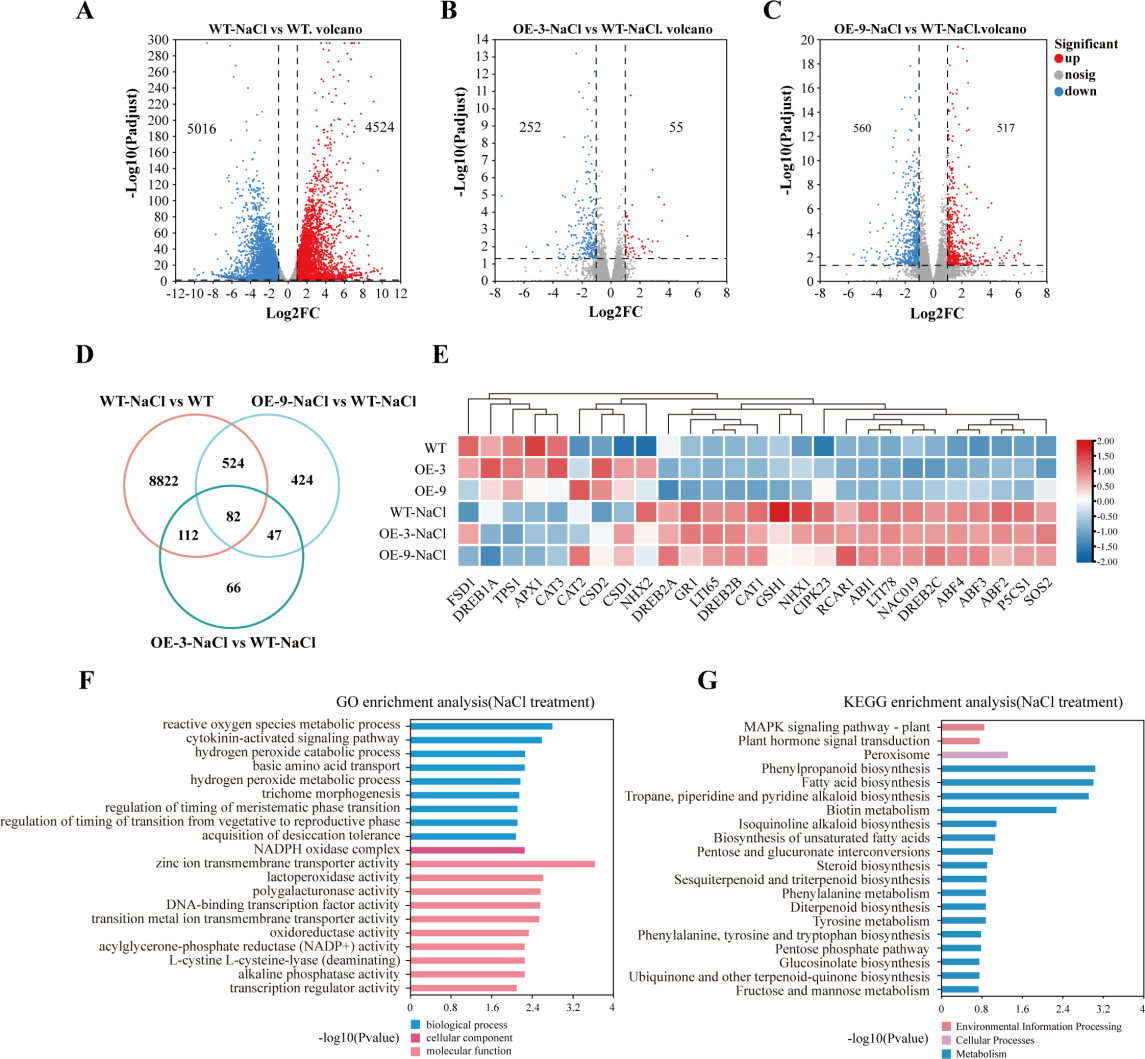
Transcriptome analysis of *BbHDA6* transgenic *Arabidopsis thaliana* under salt stress. **(A–C)** Volcano plots of DEGs in transgenic *Arabidopsis* in response to salt stress; **(D)** Venn analysis of *BbHDA6*-mediated DEGs in response to salt stress; **(E)** Heatmap of *BbHDA6*-Mediated Salt-Responsive Gene Expression; **(F)** GO analysis of *BbHDA6*-mediated salt-responsive genes; **(G)** KEGG analysis of *BbHDA6*-mediated salt-responsive genes.

To explore *BbHDA6*’s regulatory role in salt stress, we performed transcriptome analysis on seedlings from WT *Arabidopsis* and two *BbHDA6*-transgenic lines (OE-3, OE-9) under salt treatment. In WT, 9540 salt stress-responsive genes were identified, with 1379 showing differential expression between WT and transgenic lines after NaCl treatment (**Figures 8A–C**). Of these 1379 genes, 82 overlapped and were classified as *BbHDA6*-regulated salt-responsive genes (**Figure 8D**); their expression heatmap is presented in **Supplementary Figure 6C**. GO enrichment analysis showed these genes were significantly enriched in biological processes such as zinc ion transmembrane transporter activity and reactive oxygen species (ROS) metabolism (**Figure 8F**). KEGG enrichment analysis showed DEGs were enriched in phenylpropanoid biosynthesis, fatty acid biosynthesis, tropane alkaloid biosynthesis, and biotin metabolism (**Figure 8G**). Key salt stress-responsive genes (*SOS2*, *P5CS1*, *NHX2*, *DREB2A*, *CSD2*, *CAT3*) were differentially expressed between WT and transgenic lines after NaCl treatment (**Figure 8E**), confirming *BbHDA6* participates in plant salt stress responses. Furthermore, key genes regulating seed germination and root elongation (*LEA14*, *ABI5*, *PYR1*, *CYP707A1*, *CYCD3;1*, *PCNA1*) were differentially expressed under salt stress (**Supplementary Figure 6D**), indicating they are key factors for *BbHDA6*-mediated adaptive growth under salt stress.

### 3.8 Overexpression of *BbHDA6* modulates the flavonoid biosynthesis pathway in *Arabidopsis* and *N. benthamiana*

**Figure 9:**
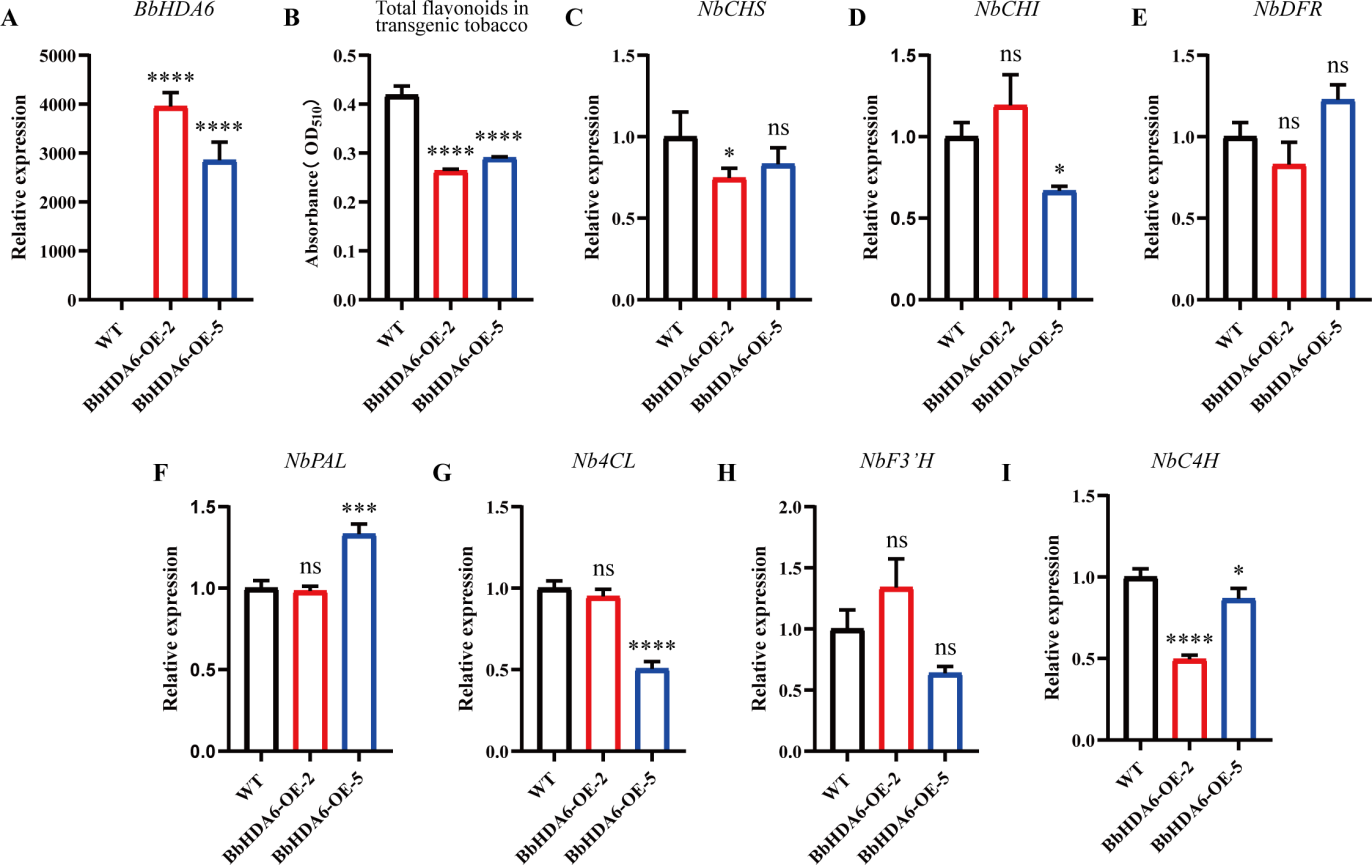
Determination of total flavonoid content and key gene expression levels in *BbHDA6* transgenic *Nicotiana benthamiana*. **(A)** Relative expression analysis of *BbHDA6* in transgenic *N. benthamiana* lines; **(B)** Total flavonoid content in leaves of *BbHDA6* transgenic *N. benthamiana*; **(C–I)** Relative expression levels of *NbCHS*, *NbCHI*, *NbDFR*, *NbPAL*, *Nb4CL*, *NbF3’H*, and *NbC4H* in *BbHDA6* transgenic *N. benthamiana*. Statistical analysis was performed using one-way ANOVA followed by Dunnett’s test, Asterisks indicate significant differences compared with the wild type: *P < 0.05, ***P < 0.001, ****P < 0.0001.

We analyzed the effects of *BbHDA6* on flavonoid biosynthesis in its transgenic *Arabidopsis* and *N. benthamiana* lines. In *BbHDA6*-transgenic *Arabidopsis*, RNA-seq identified 353 *BbHDA6*-regulated responsive genes (**Supplementary Figure 6A**). KEGG enrichment analysis showed these genes were enriched in the top 20 secondary metabolite biosynthesis pathways, including flavonoid (ko00941), anthocyanin (ko00942), and flavone and flavonol (ko00944) biosynthesis. Enriched genes included key pathway regulators such as *AT5MAT*, *LDOX*, *TT7 (F3’H)*, *TT4 (CHS)*, *DFR*, *UGT75C1*, *UF3GT*, and *UGT78D4*, and a heatmap confirmed their expression was negatively regulated by *BbHDA6* (**Supplementary Figure 7**). n transgenic N. tabacum, leaf total flavonoid content was significantly reduced by 29.72%–37.18% compared to WT, with the order of WT > *BbHDA6*-OE-5 > *BbHDA6*-OE-2 (**Figures 9A, B**; **Supplementary Figure 8**). Unlike transgenic *Arabidopsis*, *BbHDA6* exerted distinct effects on seven key *N. benthamiana* flavonoid biosynthesis genes (*NbCHS*, *NbC4H*, *NbDFR*, *NbCHI*, *NbF3’H*, *Nb4CL*, *NbPAL*). The expressions of *NbCHS* and *NbC4H* were significantly downregulated in *BbHDA6*-OE-2, as were *NbCHI*, *Nb4CL*, and *NbC4H* in *BbHDA6*-OE-5. By contrast, the expression of *NbPAL* in *BbHDA6*-OE-5 was markedly upregulated. In *BbHDA6*-OE-2, *NbCHI*, *NbPAL*, and *Nb4CL*, as well as *NbCHS* in *BbHDA6*-OE-5, *NbDFR*, and *NbF3’H* in both transgenic lines showed expression levels similar to those in WT (**Figures 9C–I**). Collectively, *BbHDA6* is involved in plant flavonoid biosynthesis and retains its conserved role as a negative regulator.

## 4 Discussion

### 4.1 Sequence conservation and expression characteristics of *BbHDA6*

The sequence characteristics and subcellular localization of *BbHDA6* lay a molecular foundation for its functional analysis; bioinformatics results show its encoded protein belongs to the HDAC superfamily, harbors a highly conserved Class I HDAC core domain (RPD3/HDA1 subfamily histone deacetylase), shares high sequence similarity with HDA6 homologs from closely related species (e.g., *Erigeron canadensis* EcHDA6, *Tanacetum cinerariifolium* TcHDA6), and has the closest evolutionary relationship with these Asteraceae species, indicating its conserved epigenetic regulatory traits (Saradadevi et al., 2023). The nuclear localization of BbHDA6 aligns with the mechanism by which histone deacetylases regulate chromatin status to modulate gene transcription—an essential prerequisite for HDAC-mediated epigenetic regulation (Earley et al., 2006; Kurita et al., 2019)—as exemplified by *Arabidopsis thaliana* HDA6, which forms nuclear complexes with chaperone proteins to drive histone deacetylation and alter gene expression/chromatin structure (Feng et al., 2021; Liu et al., 2012; Xu et al., 2022; Yang et al., 2020; Zhou et al., 2021). Notably, BbHDA6 lacks signal peptides and transmembrane domains, with a secondary structure dominated by random coils and α-helices—features that may confer structural flexibility for interactions with transcription factors and chromatin-related proteins, providing clues for future characterization of its regulatory complex. Tissue-specific expression analysis reveals *BbHDA6* is most highly expressed in the upper leaves of *B. balsamifera*, the primary site of flavonoid accumulation, and this spatial correspondence between gene expression and flavonoid synthesis suggests an inherent link between *BbHDA6* regulation and flavonoid accumulation.

### 4.2 *BbHDA6* mediates abiotic stress responses with functional conservation

By uncovering the specific response patterns of *BbHDA6* to four abiotic stresses (low temperature, drought, salt, and ABA), this study confirmed its functional conservation with the *Arabidopsis* homolog *AtHDA6* in stress regulation (Chen et al., 2010; Chen and Wu, 2010; Su et al., 2025). Distinct divergence was observed in the expression dynamics of *BbHDA6* under different stresses: its expression exhibited a short-term response characteristic of “upregulation-downregulation-recovery” under low temperature stress, indicating that this gene may participate in signal perception and transduction at the early stage of low temperature stress, and maintain intracellular environmental homeostasis through the decline of its expression level at the later stage, which is consistent with the core mechanism of *AtHDA6* in regulating low temperature response (Su et al., 2025); the transient upregulation under drought stress suggested that *BbHDA6* exerts a regulatory role in the early defense response to drought stress, probably affecting the transcription of downstream stress-resistant genes through histone deacetylation modification (Li et al., 2023); the fluctuating expression under salt stress implied that it can adapt to the complex cellular signaling network under salt stress through dynamic expression regulation, so as to cope with the dynamic environmental changes during the stress process (Chen et al., 2010); while the time-dependent downregulation under ABA stress demonstrated that *BbHDA6* participates in abiotic stress responses mainly through the ABA-mediated signaling pathway, reflecting the conserved regulatory position of HDA6 family genes in the ABA signaling pathway (Chen and Wu, 2010; Luo et al., 2012). These results indicated that *BbHDA6*, as a key regulatory factor in abiotic stress responses, can participate in the stress adaptation of *B. balsamifera* by modulating its own expression level, thus providing a key molecular target for elucidating the molecular mechanism of stress resistance and the genetic improvement of stress-resistant traits in *B. balsamifera*.

### 4.3 *BbHDA6* from *B. balsamifera* mediates ABA response in transgenic *Arabidopsis*

Through phenotypic analysis and transcriptome sequencing, this study confirmed that *B. balsamifera BbHDA6* is involved in the ABA response of transgenic *Arabidopsis*, with its functional regulation exhibiting concentration dependence (Chen et al., 2010; Su et al., 2021). Under normal growth conditions, there were no significant differences in seed germination rate or seedling root length between *BbHDA6*-transgenic lines and wild-type (WT) *Arabidopsis*, indicating that this gene has no obvious impact on the basic growth of *Arabidopsis*. After ABA treatment, transgenic lines showed superior seed germination, longer root lengths, and enhanced tolerance under low concentrations (0.5, 1 μM); in contrast, under high ABA concentration (5 μM), transgenic lines exhibited more germination inhibition, shorter roots, and increased sensitivity, suggesting that *BbHDA6*-mediated regulation of the ABA response is stress concentration-dependent. RNA-seq results further revealed that *BbHDA6* overexpression significantly altered the transcriptome profile of transgenic *Arabidopsis* under ABA treatment. Key genes responding to ABA signals, such as *CYP707A1*, *SnRK2.3*, and *GASA6*, were differentially expressed between WT and the two transgenic lines, which may be one of the reasons for the phenotypic differences observed in *BbHDA6*-transgenic *Arabidopsis* under ABA treatment (Fujita et al., 2009; Kushiro et al., 2004; Ma et al., 2009; Nakashima et al., 2009; Nishimura et al., 2009; Qu et al., 2016). The 157 ABA-responsive genes regulated by *BbHDA6* were significantly enriched in multiple pathways, including biosynthesis of various plant secondary metabolites, phenylpropanoid metabolism, and plant hormone signal transduction, with *BbHDA6* inhibiting the activation of most upregulated genes. The identification of these ABA-responsive genes provides a foundation for further deciphering the specific action sites of *BbHDA6* in the ABA signaling pathway.

### 4.4 *BbHDA6* is involved in the response to osmotic stress

This study revealed that *BbHDA6* exerts a positive role in enhancing osmotic stress tolerance in *Arabidopsis* under mannitol-induced osmotic stress mimicking drought conditions. Phenotypically, overexpression of *BbHDA6* generally promoted primary root elongation under various concentrations of mannitol and significantly reduced the incidence of seedling growth abnormalities under 200 mM mannitol, indicating that *BbHDA6* positively regulates adaptation to osmotic stress. Notably, seed germination exhibited a distinct dynamic characterized by “initial inhibition followed by promotion”. Under 100~200 mM mannitol, transgenic lines initiated germination slightly more slowly than the wild type at 1~2 days, but achieved significantly higher germination rates at 5~7 days. This pattern suggests that *BbHDA6* may delay germination onset to allow more sufficient stress preparation, thereby conferring subsequent growth advantages. Under high−concentration (300 mM) mannitol treatment, stress tolerance varied among transgenic lines. The high−expression line OE−3 showed delayed germination but the most stable seedling growth, indicating that the regulatory effect of *BbHDA6* depends on a stress−concentration threshold. Based on the regulatory role of *BbHDA6* in key flavonoid biosynthetic genes described in Section 3.8, we propose that under mannitol−simulated drought stress, *BbHDA6* may trigger a unique metabolic shift and transcriptional reprogramming through epigenetic modifications. It may redirect metabolic precursors toward the synthesis of other protective compounds such as osmoprotectants (Bharti et al., 2015; Bulgakov et al., 2024; To et al., 2011), thereby enhancing stress resistance in transgenic *Arabidopsis*. Furthermore, the functionality of *BbHDA6* overexpression under mannitol treatment is dependent on both stress intensity and its own expression level (Chen et al., 2010).

### 4.5 *BbHDA6* is involved in salt stress response of transgenic *Arabidopsis*

Phenotypic and transcriptome analyses demonstrate *BbHDA6* mediates salt stress responses in transgenic *Arabidopsis* with expression level-dependent and stage-specific effects. At the germination stage, high-expression line OE-3 maintains stable germination advantages across NaCl concentrations, while low-expression line OE-9 shows transient advantages only at specific time points/concentrations—indicating germination promotion depends on *BbHDA6* expression levels (Chen et al., 2010). For seedling root growth, salt stress inhibits elongation: transgenic lines have significantly shorter roots than WT under 150 and 200 mM NaCl, with OE-9 showing the shortest roots at 100 mM and a higher proportion of abnormally growing plants at this concentration—revealing growth stage-specific regulation of germination, root development, and salt tolerance. Transcriptome data confirm differential expression of key salt-responsive and growth-regulating genes between WT and transgenic lines, supporting *BbHDA6*-mediated salt stress responses. Additionally, comparison with ABA-induced KEGG enrichment pathways shows *BbHDA6*-regulated salt-responsive genes are enriched in the phenylpropane synthesis pathway (a core upstream pathway of flavonoid synthesis), suggesting *BbHDA6* integrates ABA and salt stress signals via spatiotemporal regulation of flavonoid biosynthesis—emerging as a core molecular pathway for *BbHDA6*-mediated abiotic stress responses (Brunetti et al., 2018; Bulgakov et al., 2024; Chen et al., 2010; Daryanavard et al., 2023; Gallusci et al., 2023; Lämke and Bäurle, 2017).

### 4.6 Conserved negative regulatory role of *BbHDA6* in mediating plant flavonoid biosynthesis

Through heterologous expression of *BbHDA6* in *Arabidopsis* and *N. benthamiana*, this study showed that *BbHDA6* participates in plant flavonoid biosynthesis and retains core negative regulatory function. In transgenic *Arabidopsis*, 353 *BbHDA6*-regulated genes, identified via RNA sequencing, were significantly enriched via KEGG analysis in secondary metabolic pathways including flavonoid and anthocyanin biosynthesis. Key flavonoid biosynthesis genes (e.g., *AT5MAT* and *LDOX*) were negatively regulated by *BbHDA6*, consistent with the conserved regulatory properties of HDACs (Su et al., 2021; Zeng et al., 2025). In transgenic *N. benthamiana*, *BbHDA6* overexpression significantly reduced leaf total flavonoid content by 29.72%–37.18% compared to the wild type, further confirming its negative regulatory role. However, it exhibited line-specific differences in regulating seven key flavonoid biosynthesis genes, distinct from the unified negative regulatory pattern in *Arabidopsis*. This apparent discrepancy between the two heterologous expression systems can be attributed to the inherent divergence of transcriptional regulatory networks between plant species. As a histone deacetylase, the regulatory behavior of *BbHDA6* depends on specific interacting proteins, target chromatin microenvironments, and upstream/downstream regulatory modules present in the host plant (Chen and Wu, 2010; Huang et al., 2026; Sun et al., 2023). The distinct transcription factor complexes, promoter epigenetic landscapes, and metabolic feedback mechanisms between *Arabidopsis* and *N. benthamiana* may lead to differential recruitment or regulatory output of *BbHDA6*, resulting in species-specific and even line-specific transcriptional effects on individual flavonoid biosynthetic genes (e.g., *NbPAL*) (Chen et al., 2020; Liu et al., 2014; Ma et al., 2013). Despite these individual gene-level differences, the overall negative regulatory effect of *BbHDA6* on total flavonoid accumulation was conserved in both systems. These interspecific differences reflect the context-dependent regulatory characteristics of epigenetic modifiers under heterologous expression, rather than a loss of core function. The conserved negative regulation of *BbHDA6* on flavonoid biosynthesis provides a theoretical basis for in-depth analysis of flavonoid biosynthesis epigenetic regulation in *B. balsamifera* and a candidate gene for enhancing plant flavonoid content via *BbHDA6* expression modulation. The present study did not verify the direct binding targets of *BbHDA6*, which could be further identified using ChIP assays in future research.

## 5 Conclusion

In this study, we functionally characterized the histone deacetylase gene *BbHDA6* from *B. balsamifera*. We found that *BbHDA6* acts as a conserved negative regulator of flavonoid biosynthesis and participates in plant responses to abiotic stresses such as ABA, osmotic, and salt stress in transgenic plants. Additionally, *BbHDA6* orchestrates transcriptional reprogramming mainly associated with phenylpropanoid metabolism, secondary metabolite biosynthesis, and stress signal transduction pathways. Collectively, these findings indicate that *BbHDA6* serves as a critical epigenetic hub that integrates flavonoid metabolic homeostasis and abiotic stress adaptation. In turn, this work provides a valuable gene target and theoretical basis for the genetic improvement of medicinal plant quality and stress tolerance, further enriching our understanding of epigenetic regulation in medicinal Asteraceae species.

## Data Availability

All data generated or analyzed during this study are included within this article and the accompanying **Supplementary Files**. The RNA sequencing datasets analyzed in this study are available from the NCBI public repository (SRA accession number: PRJNA1421045).

## Supplementary material

Supplementary Figure 1 Cloning of the *BbHDA6* gene and identification of transgenic *Arabidopsis*.

Supplementary Figure 2 Bioinformatics analysis of *BbHDA6*.

Supplementary Figure 3 Motif analysis of *BbHDA6* homologous genes.

Supplementary Figure 4 Analysis of *BbHDA6* expression levels in different transgenic *Arabidopsis*.

Supplementary Figure 5 Growth analysis of *BbHDA6* transgenic *Arabidopsis* on ABA, NaCl, and mannitol-stressed media.

Supplementary Figure 6 Heatmap of *BbHDA6*-mediated DEG expression under different treatments.

Supplementary Figure 7 Transcriptome analysis of *BbHDA6* transgenic *Arabidopsis*.

Supplementary Figure 8 Standard curve for the determination of total flavonoids in *N. benthamiana*.

Supplementary Table S1 Cloning primers for *BbHDA6*.

Supplementary Table S2 RT-qPCR primers for key flavonoid synthesis genes in transgenic *N. benthamiana*.

Supplementary Table S3 Seed germination rate significance analysis among four genotypes in 1/2 MS medium.

Supplementary Table S4 Seed germination rate significance analysis among four genotypes under 0.5 μM ABA stress.

Supplementary Table S5 Seed germination rate significance analysis among four genotypes under 1 μM ABA stress.

Supplementary Table S6 Seed germination rate significance analysis among four genotypes under 5 μM ABA stress.

Supplementary Table S7 Seed germination rate significance analysis among four genotypes under 100 mM mannitol stress.

Supplementary Table S8 Seed germination rate significance analysis among four genotypes under 200 mM mannitol stress.

Supplementary Table S9 Seed germination rate significance analysis among four genotypes under 300 mM mannitol stress.

Supplementary Table S10 Seed germination rate significance analysis among four genotypes under 100 mM NaCl stress.

Supplementary Table S11 Seed germination rate significance analysis among four genotypes under 150 mM NaCl stress.

Supplementary Table S12 Seed germination rate significance analysis among four genotypes under 200 mM NaCl stress.
