# Supplementary material for: Blumea balsamifera BbHDA6 regulates abiotic stress responses and flavonoid biosynthesis in transgenic plants
Source: Front Plant Sci. 2026 Apr 22;17:1811058. doi: 10.3389/fpls.2026.1811058 (PMC13143846; doi:10.3389/fpls.2026.1811058)
Supplement: Supplementary Figure 1 — Cloning of the BbHDA6 gene and identification of transgenic Arabidopsis. [file DataSheet1.docx]

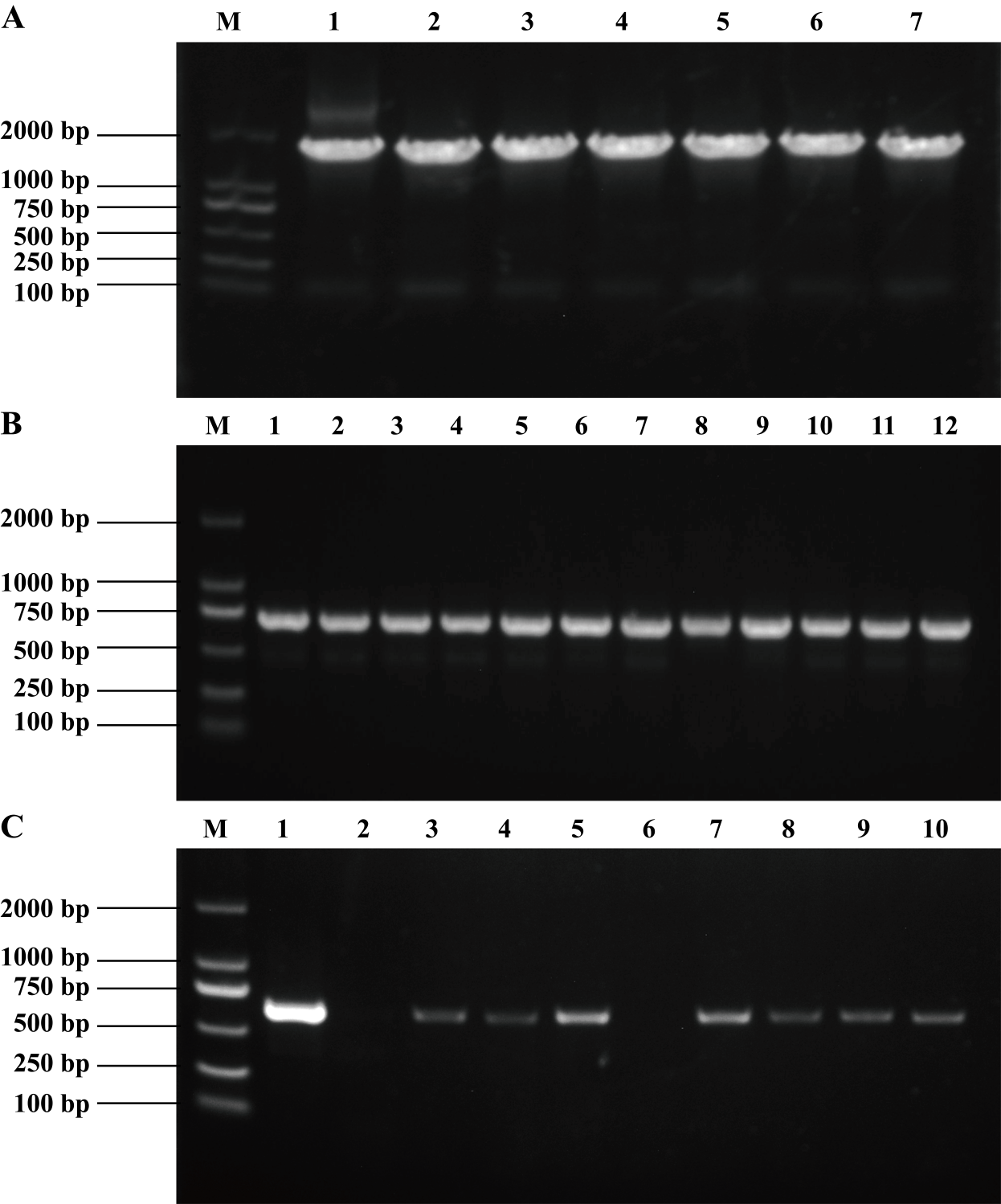


**Figure 1. Cloning of the *BbHDA6* gene and identification of transgenic *Arabidopsis***. A: Identification of BbHDA6 gene cloning; 1: Positive control; 2~7: Positive clones; B: Identification of empty vector pBGOL-0017 transformation; 1: Positive control; 2~12: Positive transformants; C: PCR identification of BbHDA6 transgenic *Arabidopsis*; 1: Positive control; 2, 6: Negative plants; 3~5, 7~10: BbHDA6-positive transgenic plants.

**
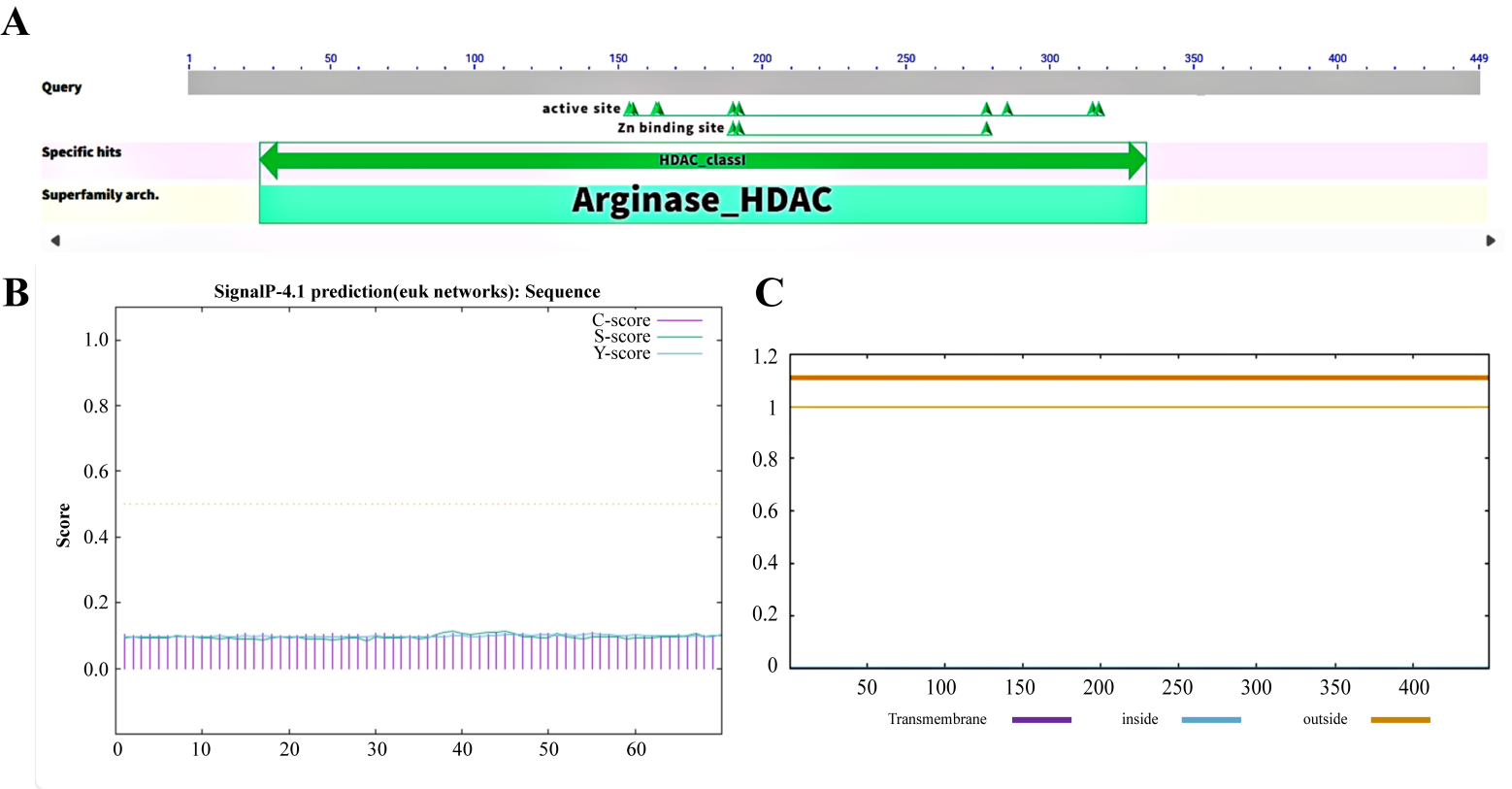
**

**Figure 2. Bioinformatics analysis of *BbHDA6*.** A: Conserved domains; B: Signal peptide; C: Transmembrane region.


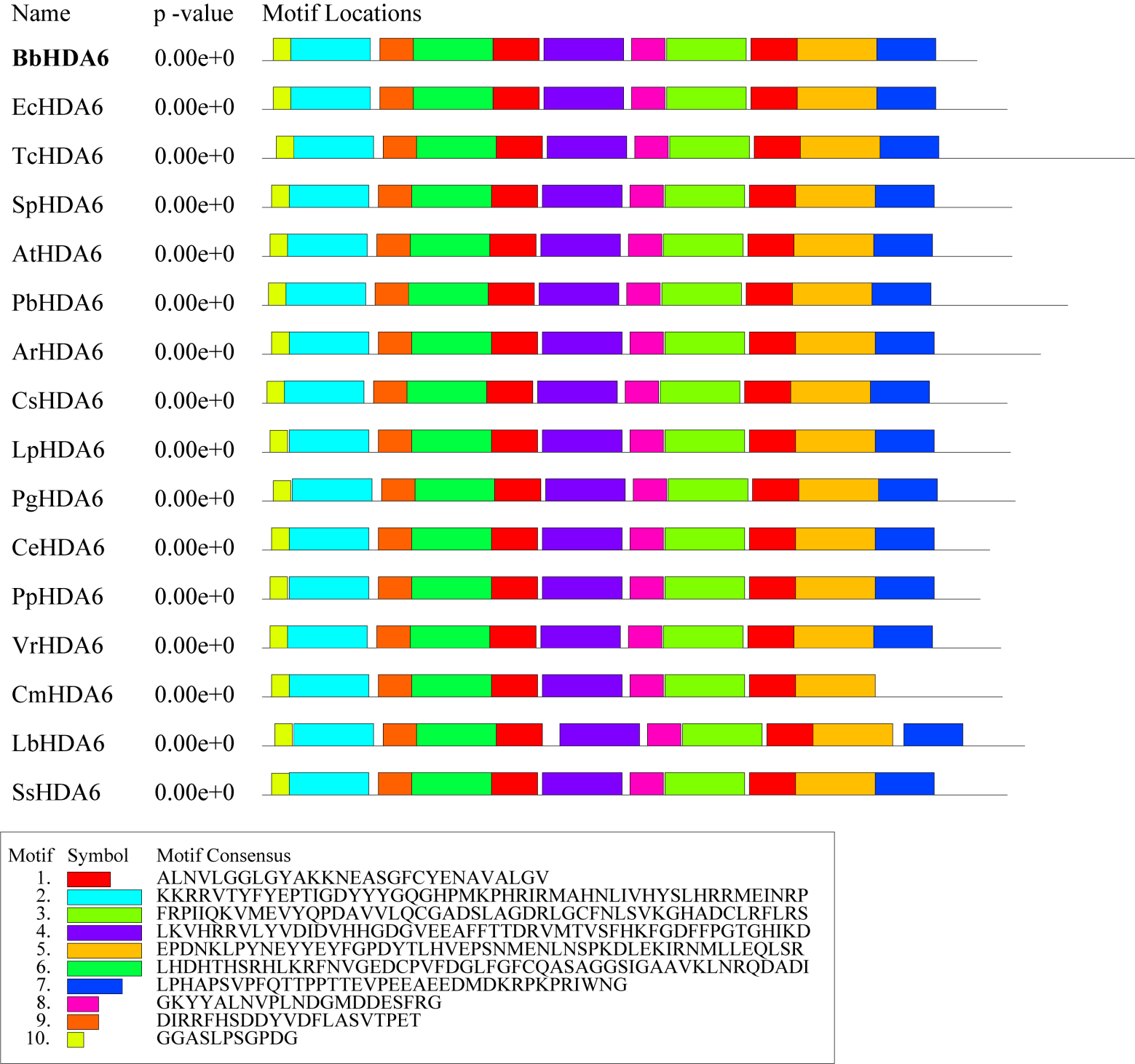


**Figure 3. Motif analysis of *BbHDA6* homologous genes**

**Figure 4. Analysis of *BbHDA6* expression levels in different transgenic *Arabidopsis.*** Statistical significance analysis was performed using one-way ANOVA combined with Dunnett's test to compare the expression levels of *BbHDA6* in each transgenic *Arabidopsis* line with the wild-type (WT).

**
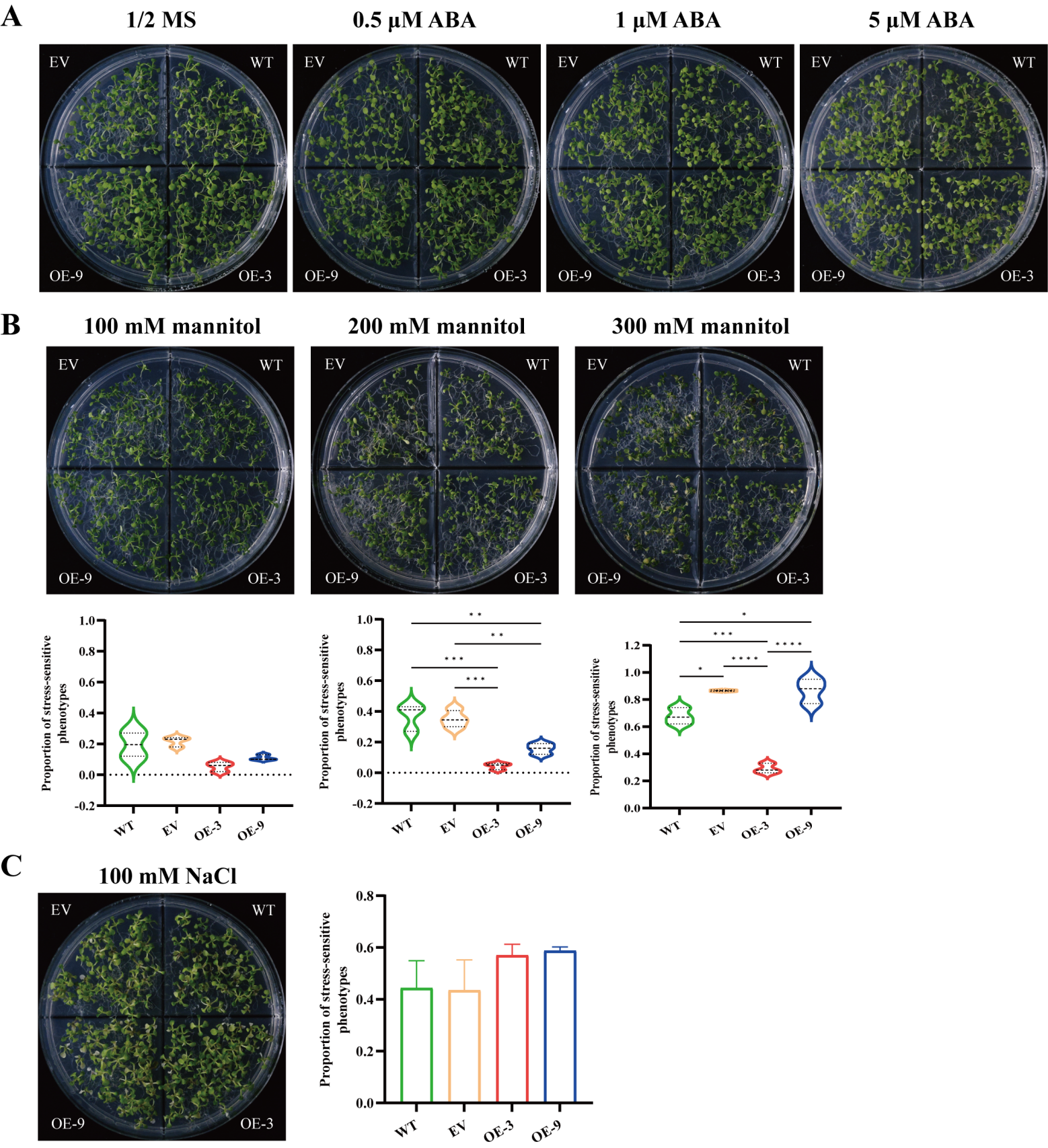
**

**Figure 5. Growth analysis of *BbHDA6* transgenic *Arabidopsis* on ABA, NaCl, and mannitol-stressed media.** A: Growth phenotypes under ABA treatment; B: Growth phenotypes under mannitol treatment; C: Growth phenotypes under NaCl treatment. Box plots and bar charts show the proportion of abnormally grown plants. Significant differences in the percentage of abnormally growing plants among different stress treatments were analyzed by one-way ANOVA followed by Tukey’s test, **P* < 0.05, ***P* < 0.01, ****P* < 0.001, *****P* < 0.0001.

**
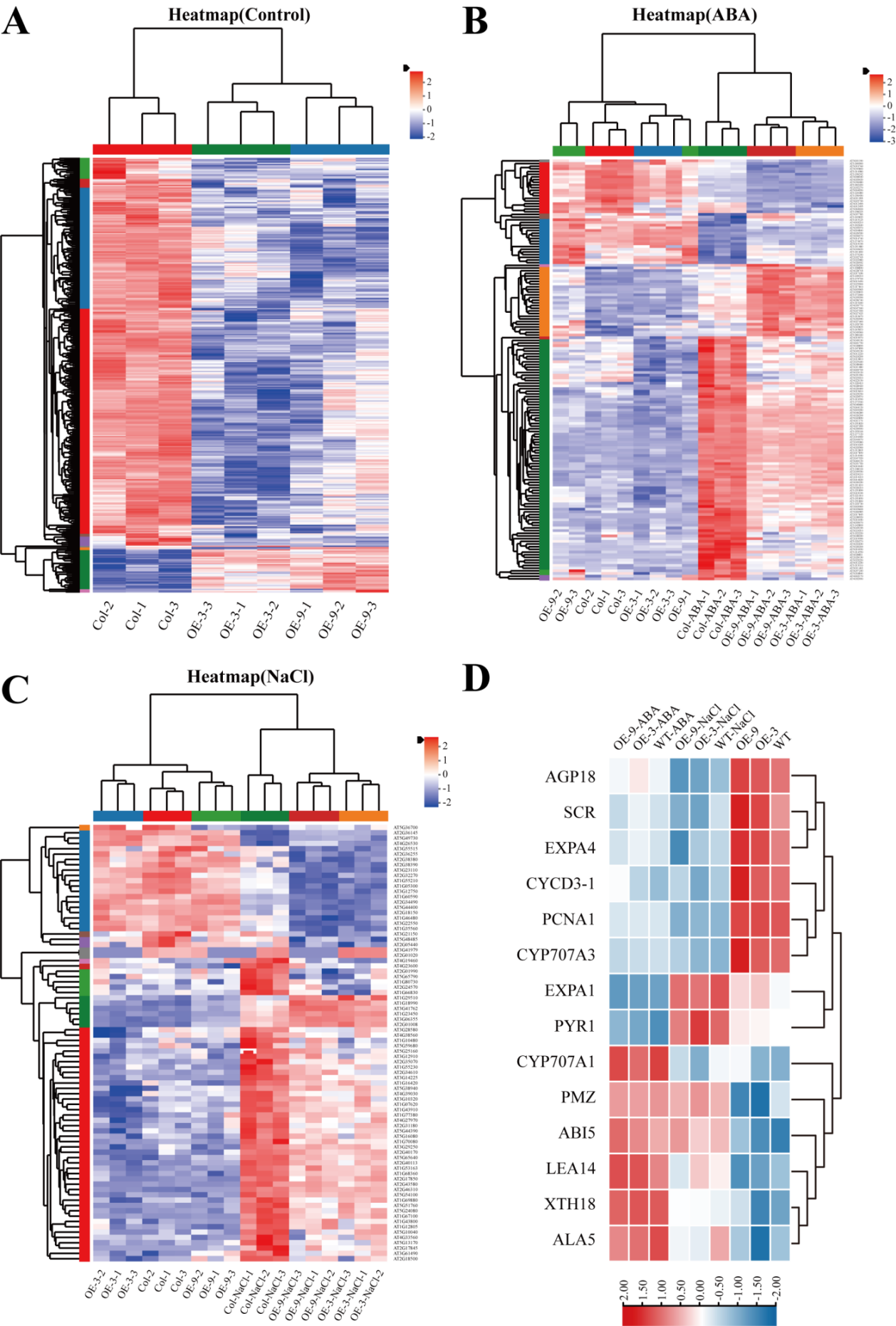
**

**Figure 6. Heatmap of *BbHDA6*-mediated DEG expression under different treatments.** A: Heatmap of *BbHDA6*-mediated DEGs in responses under normal culture conditions; B: Heatmap of *BbHDA6*-mediated ABA-responsive gene expression; C: Heatmap of *BbHDA6*-mediated salt-responsive gene expression; D: Heatmap of *BbHDA6*-mediated expression of key genes for seed germination and root growth.

**
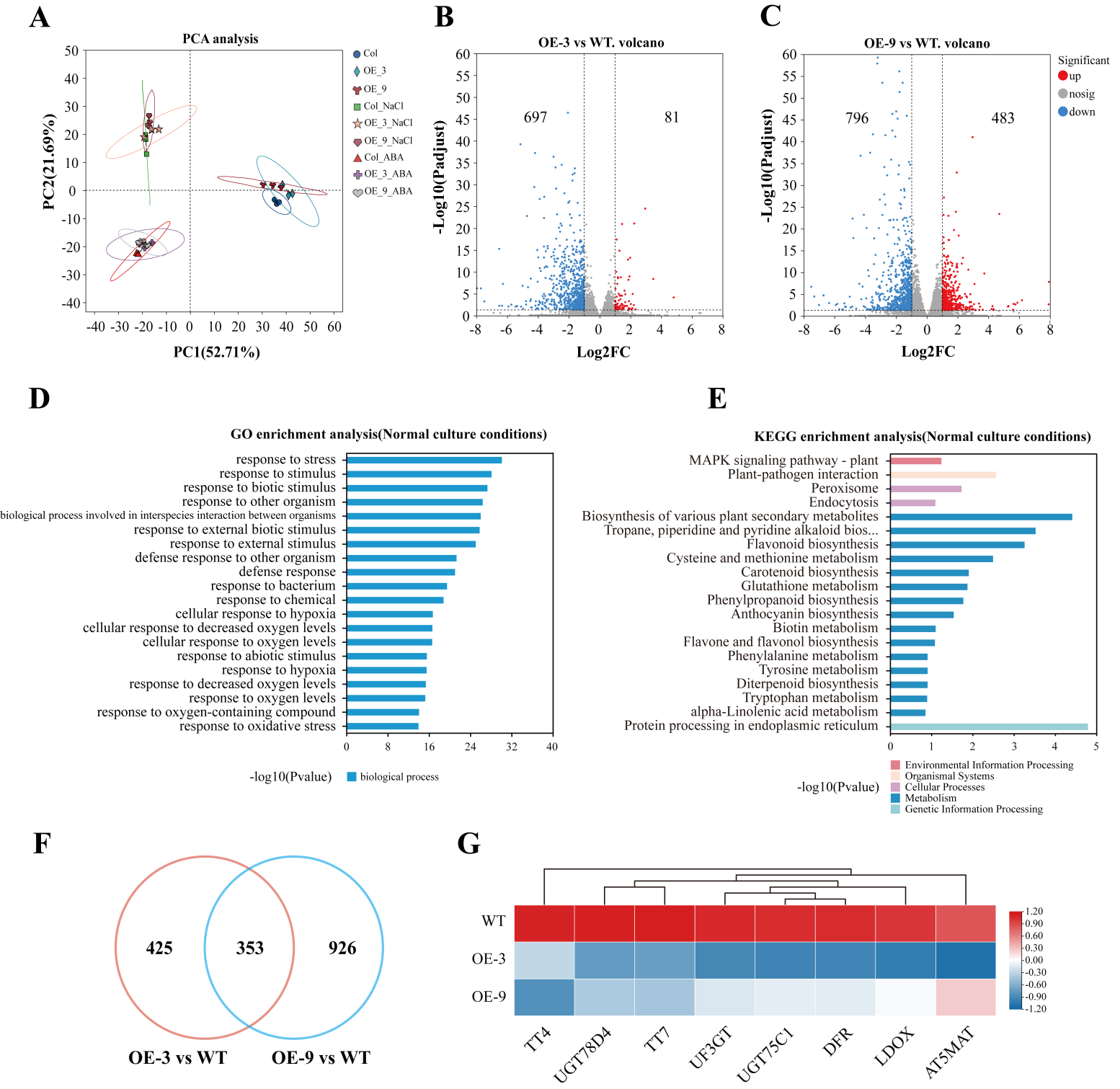
**

**Figure 7. Transcriptome analysis of *BbHDA6* transgenic *Arabidopsis*.** A: PCA analysis of tested samples; B~C: Volcano plots of *BbHDA6*-regulated DEGs; D: GO analysis of *BbHDA6*-regulated DEGs; E: KEGG analysis of *BbHDA6*-regulated DEGs; F: Venn analysis of *BbHDA6*-regulated DEGs; G: Heatmap of *BbHDA6*-mediated expression of key genes for flavonoid synthesis.

**

**

**Figure 8. Standard curve for the determination of total flavonoids in *N. benthamiana***
