# Supplementary material for: Blumea balsamifera BbHDA6 regulates abiotic stress responses and flavonoid biosynthesis in transgenic plants
Source: Front Plant Sci. 2026 Apr 22;17:1811058. doi: 10.3389/fpls.2026.1811058 (PMC13143846; doi:10.3389/fpls.2026.1811058)
Supplement: Supplementary Table S1 — Cloning primers for BbHDA6. [file Table1.docx]

**Table S1. Cloning primers for *BbHDA6***

| Primer Name | Primer Sequence (5’-3’) | Primer Purpose |
| --- | --- | --- |
| BbHDA6-F | TCGAATTCCAACAGGAGCAGGGTCCTTTG | CDS Amplification |
| BbHDA6-R | TTACCTTGTACTATTATTATTTGCATTAAAG |  |
| B6v-F | TTCGAGCTCGGTACCCGGG | Vector Construction |
| B6v-R | CCTCGAGGGATCCCCCGGG |  |
| B6-EcoF | AATTGAATTCATGGTTGAGTCGTCCTCCGTCG |  |
| B6-SmR | GGGCCTTGTACTATTATTATTTGCATTAAAGCTTTGAC |  |
| 1305-B6F | CGGAGCTAGCTCTAGA |  |
| 1305-B6R | GGATCCATGGTGAGCA |  |
| B6-BamF | AATTGGATCCATGGTTGAGTCGTCCTCCGTCG |  |
| B6-BamR | GCTAGGATCCCCTTGTACTATTATTATTTGCATTA |  |
| EF1a-F | AGAACGTTGCTGTTAAGGAT | *Blumea balsamifera* Reference Gene |
| EF1a-R | GCATAACCGTTTCCAATCTG |  |
| Actin-F | GGTACCGGTATGGTGAAGGC | *Arabidopsis* Reference Gene |
| Actin-R | TGCATCCTTCTGGTTCATCCC |  |
| B6-qF | GACAGGGTGATGACGGTCTCTTTTC | BbHDA6 RT-qPCR Identification Primer |
| B6-qR | GGTCTGAACAGACCACGAAAGCTATC |  |
| B6Fv-R | CATGCGGATTCGATGGGGTTTCATTG | *BbHDA6* Transformation Identification Primer |
| Fv-F | GAGACTTTTCAACAAAGGGTAATATCGG |  |

**Table S2. RT-qPCR primers for key flavonoid synthesis genes in transgenic *N. benthamiana***

| Primer Name | Primer Sequence (5’-3’) | Description |
| --- | --- | --- |
| NbCHS-F | CTAGTGGAGTGGACATGCCC | RT-qPCR |
| NbCHS-R | CCTTAGCCAGTCGGAGAACA |  |
| NbC4H-F | CACTGGAAGAAACCCGAAGA |  |
| NbC4H-R | CAACGCCAAACGGAAGATAT |  |
| NbDFR-F | ATGTGGCAAAGATGGTCCG |  |
| NbDFR-R | AAGGGAAGCAACTGTTTCTGTC |  |
| NbCHI-F | CTGTCTTCTCTCGCCGCTAAATG |  |
| NbCHI-R | GCCACCTTCTCTGAGTATTGCTTAC |  |
| NbF3’H-F | GCCAGACAAACCAGAAGGATG |  |
| NbF3’H-R | TCAGGCTGTGGACACTTTGG |  |
| Nb4CL-F | ACTGGCGACATTGGGTTCA |  |
| Nb4CL-R | ACTTCTCCGGCTTGCTCATC |  |
| NbPAL-F | GACCTAATGGTGAGACCCTGAA |  |
| NbPAL-R | AGAAAAGAGCGCGAGAATGTT |  |
| NbEF1α-F | AGAGGCCCTCAGACAAAC | *N. benthamiana* reference gene |
| NbEF1α-R | TAGGTCCAAAGGTCACAA |  |

**Table S3. Seed germination rate significance analysis among four genotypes in 1/2 MS medium**

| 1/2 MS | 1 | 2 | 3 | 4 | 5 | 6 | 7 |
| --- | --- | --- | --- | --- | --- | --- | --- |
| EV | ns | ns | ns | ns | ns | ns | ns |
| OE-3 | ns | ns | ns | ns | ns | ns | ns |
| OE-9 | ns | ns | ns | ns | ns | ns | ns |

**Note:** Two-way analysis of variance (ANOVA) coupled with Dunnett’s multiple comparisons tests was performed to assess significant differences in seed germination rates of *Arabidopsis* with different genotypes across various stress treatment days, relative to the wild-type (WT). Statistical significance was indicated as: *P* < 0.05 (*), *P* < 0.01 (**), *P* < 0.001 (***), and *P* < 0.0001 (****). The same data processing approach was applied to all data presented in Tables S4~S12.

**Table S4. Seed germination rate significance analysis among four genotypes under 0.5 μM ABA stress**

| 0.5uM ABA | 1 | 2 | 3 | 4 | 5 | 6 | 7 |
| --- | --- | --- | --- | --- | --- | --- | --- |
| EV | ns | ns | ns | ns | ns | ns | ns |
| OE-3 | ns | ns | ns | ns | **** | ** | ns |
| OE-9 | ns | ns | ns | ns | **** | **** | ns |

**Table S5. Seed germination rate significance analysis among four genotypes under 1 μM ABA stress**

| 1uM ABA | 1 | 2 | 3 | 4 | 5 | 6 | 7 |
| --- | --- | --- | --- | --- | --- | --- | --- |
| EV | ns | ns | ns | ns | ns | ns | ns |
| OE-3 | ns | ns | ns | **** | **** | * | * |
| OE-9 | ns | ns | *** | ns | **** | ns | ns |

**Table S6. Seed germination rate significance analysis among four genotypes under 5 μM ABA stress**

| 5uM ABA | 1 | 2 | 3 | 4 | 5 | 6 | 7 |
| --- | --- | --- | --- | --- | --- | --- | --- |
| EV | ns | ns | ns | ns | ns | ns | * |
| OE-3 | ns | ns | ns | ** | ** | **** | ns |
| OE-9 | ns | ns | ns | ns | ns | ns | * |

**Table S7. Seed germination rate significance analysis among four genotypes under 100 mM mannitol stress**

| 100 mM mannitol | 1 | 2 | 3 | 4 | 5 | 6 | 7 |
| --- | --- | --- | --- | --- | --- | --- | --- |
| EV | ns | * | ns | ns | ns | ns | ns |
| OE-3 | ns | *** | ns | ns | ns | ns | ns |
| OE-9 | ns | **** | ns | ns | ns | ns | ns |

**Table S8. Seed germination rate significance analysis among four genotypes under 200 mM mannitol stress**

| 200 mM mannitol | 1 | 2 | 3 | 4 | 5 | 6 | 7 |
| --- | --- | --- | --- | --- | --- | --- | --- |
| EV | ns | ns | ns | ns | ns | ns | ns |
| OE-3 | ns | **** | ns | ns | ns | ns | ns |
| OE-9 | * | **** | ns | ns | ns | ns | ns |

**Table S9. Seed germination rate significance analysis among four genotypes under 300 mM mannitol stress**

| 300 mM mannitol | 1 | 2 | 3 | 4 | 5 | 6 | 7 |
| --- | --- | --- | --- | --- | --- | --- | --- |
| EV | ns | ns | ns | ns | ns | ns | ns |
| OE-3 | ns | ns | **** | **** | **** | **** | ** |
| OE-9 | ns | ns | **** | **** | ns | ns | ns |

**Table S10. Seed germination rate significance analysis among four genotypes under 100 mM NaCl stress**

| 100mM NaCl | 1 | 2 | 3 | 4 | 5 | 6 | 7 |
| --- | --- | --- | --- | --- | --- | --- | --- |
| EV | ns | ns | ns | ns | ns | ns | ns |
| OE-3 | ns | **** | **** | **** | **** | **** | **** |
| OE-9 | ns | ns | ns | * | ns | ns | ns |

**Table S11. Seed germination rate significance analysis among four genotypes under 150 mM NaCl stress**

| 150mM NaCl | 1 | 2 | 3 | 4 | 5 | 6 | 7 |
| --- | --- | --- | --- | --- | --- | --- | --- |
| EV | ns | ns | ns | ns | ns | ns | ns |
| OE-3 | ns | ns | **** | **** | **** | * | ** |
| OE-9 | ns | ns | ns | *** | ns | ns | ns |

**Table S12. Seed germination rate significance analysis among four genotypes under 200 mM NaCl stress**

| 200mM NaCl | 1 | 2 | 3 | 4 | 5 | 6 | 7 |
| --- | --- | --- | --- | --- | --- | --- | --- |
| EV | ns | ns | ns | ns | ns | ns | ns |
| OE-3 | ns | ns | ** | **** | **** | **** | ns |
| OE-9 | ns | ns | ns | ns | * | ns | ns |
